# What Works for Whom in School-Based Anti-bullying Interventions? An Individual Participant Data Meta-analysis

**DOI:** 10.1007/s11121-022-01387-z

**Published:** 2022-07-07

**Authors:** Maud Hensums, Brechtje de Mooij, Steven C. Kuijper, BIRC: the anti-Bullying Interventions Research Consortium, Donna Cross, Ann DeSmet, Claire F. Garandeau, Katja Joronen, Bonnie Leadbeater, Ersilia Menesini, Benedetta Emanuela Palladino, Christina Salmivalli, Olga Solomontos-Kountouri, René Veenstra, Minne Fekkes, Geertjan Overbeek

**Affiliations:** 1 Department of Child Development and Education, University of Amsterdam, Amsterdam, The Netherlands; 2 Research Priority Area Yield, University of Amsterdam, Amsterdam, The Netherlands; 3 Department of Medical Oncology, Cancer Center Amsterdam, Amsterdam University Medical Centers, University of Amsterdam, Amsterdam, The Netherlands; 4 Prevention and Health, TNO, Netherlands Organization of Applied Scientific Research, The Hague, The Netherlands; 5 Health Promotion and Education Research Team, Telethon Kids Institute, Nedlands, Australia; 6 Faculty of Medicine, Dentistry and Health Sciences, University of Western Australia, Crawley, Australia; 7 Research Center for the Promotion of Health, Prosocial Behavior and Wellbeing PACE, Faculty of Psychological and Educational Sciences, Université Libre de Bruxelles, Brussels, Belgium; 8 Department of Communication Studies, University of Antwerp, Antwerp, Belgium; 9 Department of Psychology, INVEST Research Flagship, University of Turku, Turku, Finland; 10 Department of Nursing Science, University of Turku, Turku, Finland; 11 Department of Psychology, University of Victoria, Victoria, Canada; 12 Department of Education, Interculture, Literature and Psychology, University of Florence, Florence, Italy; 13 Theological School, Church of Cyprus, Nicosia, Cyprus; 14 Department of Sociology, University of Groningen, Groningen, The Netherlands

**Keywords:** Individual participant data (IPD) meta-analysis, Anti-bullying interventions, Effectiveness, Bullying, Victimization

## Abstract

The prevalence of bullying worldwide is high (UNESCO, 2018). Over the past decades, many anti-bullying interventions have been developed to remediate this problem. However, we lack insight into for whom these interventions work and what individual intervention components drive the total intervention effects. We conducted a large-scale individual participant data (IPD) meta-analysis using data from 39,793 children and adolescents aged five to 20 years (*M*_age_ = 12.58, *SD* = 2.34) who had participated in quasi-experimental or randomized controlled trials of school-based anti-bullying interventions (i.e., 10 studies testing nine interventions). Multilevel logistic regression analyses showed that anti-bullying interventions significantly reduced self-reported victimization (*d* =  − 0.14) and bullying perpetration (*d* =  − 0.07). Anti-bullying interventions more strongly reduced bullying perpetration in younger participants (i.e., under age 12) and victimization for youth who were more heavily victimized before the intervention. We did not find evidence to show that the inclusion of specific intervention components was related to higher overall intervention effects, except for an iatrogenic effect of non-punitive disciplinary methods–which was strongest for girls. Exploratory analyses suggested that school assemblies and playground supervision may have harmful effects for some, increasing bullying perpetration in youth who already bullied frequently at baseline. In conclusion, school-based anti-bullying interventions are generally effective and work especially well for younger children and youth who are most heavily victimized. Further tailoring of interventions may be necessary to more effectively meet the needs and strengths of specific subgroups of children and adolescents.

**Supplementary Information:**

The online version contains supplementary material available at 10.1007/s11121-022-01387-z.

## Introduction 

The prevalence of bullying worldwide is high. Across continents, one in three children are bullied once or repeatedly every month (UNESCO, 2018). In some regions (Canada, Europe, and Australia), one in ten children experiences cyberbullying (UNESCO, 2018). Consequently, in many countries, bullying has been on the scientific and political agenda for some time, resulting in the development and implementation of anti-bullying interventions. Possibly, as a result, traditional bullying has declined in almost half of the countries worldwide in the past decade (UNESCO, 2018). However, not all youth benefit from anti-bullying interventions to the same extent (Smith et al., 2005). Despite positive trends, we lack insight into for whom these bullying interventions are specifically effective, and what makes them work. This knowledge is crucial to develop more effective and tailored anti-bullying programs (Ttofi & Farrington, 2009). Therefore, we conducted an individual participant data (IPD) meta-analysis to examine for whom these school-based anti-bullying interventions are more or less effective and which individual intervention components drive the effects of anti-bullying interventions.

### Consequences of Bullying and Victimization

Bullying refers to aggressive physical and relational behavior intended to harm the other that occurs repeatedly in a relationship characterized by a power imbalance (Olweus, 1993). It is one of the most common expressions of violence in the peer context (Menesini & Salmivalli, 2017) and can be detrimental for those victimized, even more so when those victimized also partake in bullying (i.e., “bully-victims”; see Arseneault et al., 2006). Victims and bully-victims are more likely to develop problems such as anxiety, loneliness, and depression (Christina et al., 2021; Reijntjes et al., 2010), have worse physiological outcomes when under social stress (Giletta et al., 2018), and are more likely to engage in suicidal ideation and suicide attempts (Van Geel et al., 2014). Engaging in bullying may also be harmful to perpetrators. Bullies are more likely to abuse substances (Ttofi et al., 2016) and become criminal offenders (Ttofi et al., 2011) and are at heightened risk for suicide ideation and attempts (Holt et al., 2015). For some, negative consequences of bullying persist well into adulthood (e.g., Sigurdson et al., 2015).

### Anti-bullying Intervention Effects

Anti-bullying interventions are designed to prevent and decrease bullying behavior. Most of these interventions are multifaceted packages that combine intervention components. Some of these intervention components focus on cognitive-emotional skills to improve bystanders’ and bullies’ emotion regulation and increase empathy for victims (e.g., Trip et al., 2015). Other components address the victims’ (and sometimes bullies’) social skills to teach them how to cope with negative feelings and situations (e.g., DeRosier, 2004). Yet other components focus on individual behaviors, group norms, and promoting a positive social climate in schools (e.g., Paluck et al., 2016).

Extensive research has examined the effects of anti-bullying interventions. Meta-analyses indicate anti-bullying interventions are moderately effective. Gaffney et al. (2019a) evaluated four anti-bullying interventions (KiVa, NoTrap!, Olweus Bullying Prevention Program, and ViSC) across 12 different countries. These interventions were found to reduce bullying perpetration by 19–20% and victimization by 15–16%. De Mooij et al. (2020a) found anti-bullying programs had a moderately strong effect (*d* = 0.67) on victimization and bullying perpetration, which is in line with another review, showing that anti-bullying interventions reduce bullying and victimization by 20–23% (Ttofi & Farrington, 2009).

### What Works for Whom?

Although generally effective, the magnitude and direction of anti-bullying intervention effects differ between interventions (Gaffney et al., 2019b). This may be due to variation in program composition and implementation of specific components (Chorpita et al., 2005), raising the question of which components are more or less effective for whom. Finding an answer to this question increases insight into what works–and what does not–and can stimulate the development of efficient, cost-effective, and tailored intervention programs (De Mooij et al., 2020a).

Anti-bullying interventions differ regarding their underlying theoretical frameworks, target populations, and components related to *anti-bullying policies or rules* and *the skills taught and practiced* (Farrington & Ttofi, 2009). Components at the policy level may require schools to adopt a school-wide anti-bullying policy, set classroom rules, use (non-)punitive disciplinary methods, increase supervision at bullying “hotspots” like playgrounds, and use peer educators. Skill-oriented components might be psychoeducation (enhancing students’ knowledge and awareness about the bullying victimization process), teaching social or cognitive-emotional skills, and psychophysical exercises to reduce victimization and bullying.

The “what works for whom” question has been posed but not yet examined as such. Rather, previous studies have examined “what works” (e.g., De Mooij et al., 2020a; Gaffney et al., 2019b, 2021; Ttofi & Farrington, 2009) or “for whom does it work” (e.g., Garandeau et al., 2014; Nocentini et al., 2019; Yeager et al., 2015) in isolation–with the former group of studies examining effects of components in the total samples, and the latter group of studies examining the effects of interventions across subgroups. For instance, a recent aggregate data meta-analysis (Gaffney et al., 2021) examined “what works” in anti-bullying interventions, showing that the presence of specific components (i.e., a whole-school approach, anti-bullying policies, classroom rules, information for parents, informal peer involvement, and work with victims) was linked to larger effect sizes for school bullying perpetration and victimization outcomes. This study provided valuable insights, but it is necessary to take the next step: harnessing the power of numbers in an individual participant data (IPD) meta-analysis by pooling data across different anti-bullying intervention studies. This is especially important with analyses that necessitate the delineation of subgroups, either in terms of interventions that have (or do not have) specific components or in terms of subgroups of children and adolescents receiving the intervention.

Assuming different subgroups of youth are differentially affected by anti-bullying interventions (De Mooij et al., 2020a; Smith et al., 2005), identifying these subgroups can help better tailor interventions to children’s and adolescents’ individual needs. In this paper, we delineate subgroups according to youth’s age, sex, ethnicity, socioeconomic status, and initial levels of victimization and bullying.

We could not develop specific hypotheses about individual components that work better for specific subgroups based on the extant literature. It was possible to develop hypotheses about whether separate components, *in general,* would work better for some. We expected that (1) children below 12 years benefited more from school-based anti-bullying intervention components than those above 12 years, based on research that showed that anti-bullying interventions become less effective from grade eight onwards and may then even induce iatrogenic effects, possibly because current anti-bullying interventions do not meet adolescence-specific needs for status and respect (Yeager et al., 2015, 2018); (2) boys benefited more from school-based anti-bullying intervention components than girls, as research has shown that interventions may gravitate towards visible, more explicit–typically male–bullying (Barbero et al., 2012; Volk et al., 2012); (3) school-based anti-bullying interventions are more effective for ethnic majority than minority youth, because interventions generally do not attend to ethnicity-related issues, such as race and ethnicity-based stereotype harassment (Peguero & Williams, 2013; Vervoort et al., 2010; Yeager et al., 2015); (4) youth with higher socioeconomic status (SES) benefited more from school-based anti-bullying interventions than youth with lower SES, as lower SES youth may need a relatively intense intervention since they might be more likely to engage in bullying or to become victimized, although this might be different across countries (Hosozawa et al., 2021; Tippett & Wolke, 2014); and (5) youth who bully or who are victimized prior to intervention benefited more from school-based anti-bullying interventions, based on research that showed intervention effects to be larger for those victimized or bullied before the intervention (e.g., Ferguson et al., 2007; Juvonen et al., 2016–for an exception, see Kaufman et al., 2018). This suggests that interventions are more relevant and engaging for youth with more severe problems, who may also have more room for behavior change.

### This Study

This study combined data from different studies on the effects of school-based anti-bullying interventions. The individual participant data (IPD) approach synthesizes individual data from randomized or quasi-experimental trials, allowing for analyzing intervention effects at the individual level instead of at the aggregate study level. Consequently, an IPD meta-analysis has greater power to test moderators and reduce potential bias compared with an aggregate data meta-analytic approach (Riley et al., 2010). Our objectives were to assess (1) the overall effect of school-based anti-bullying interventions, (2) which youth benefited more from school-based anti-bullying interventions as a whole (“for whom”), and (3) which youth benefited most from specific intervention components (‘what works for whom’).

## Methods

### Identification and Selection of Studies

We performed a systematic search in PsycINFO, Medline, Web of Science, and ERIC in January 2019. Search terms included bullying (cyberbullying or traditional bullying) combined with school-based intervention studies with a (quasi-)experimental design in primary, middle, or high schools (SupMat1). An initial title screening by SK assessed eligibility; abstracts were screened by four authors. Disagreements regarding the eligibility of a study were discussed until a consensus was reached. Finally, SK screened the full text of the remaining 95 studies.

Studies of school-based anti-bullying interventions in primary, middle, and high schools worldwide were eligible for inclusion, provided they had an experimental (i.e., randomized controlled trials) or quasi-experimental design (with at least a control group) and used a bullying and/or victimization measure before and after interventions. Studies were included if published in peer-reviewed scientific journals, focused on behavior, and included either self or peer-reported class- or school-based measures. We excluded studies when the intervention aimed to reduce other forms of aggression or harassment and did not specifically mention to target bullying–not all forms of aggression can be characterized as bullying, and interventions aimed at decreasing aggression take many different forms and have many different outcome measures (hindering successful harmonization of data). In the second phase of screening, we retrieved study data. Due to our IPD design, studies were only included if PIs were able and willing to send us the raw data. Finally, studies were only included when bullying or victimization was measured on a frequency scale for harmonization purposes.

After the first phase of screening, 41 papers, reporting on 36 unique studies, were eligible for inclusion, and the principal investigators (PIs) were contacted to request the anonymized data (see SupMat6 for more information on the eligible studies). The PIs of 13 studies (36.11%) shared their data, of which 10 studies could be included (SupMat2). For other studies, data were not shared due to ethical considerations, time constraints, and a lack of possibility or willingness to share data, or contact with the authors could not be established. PIs completed a data-sharing agreement and provided the raw individual item-level data. Data were then checked for missingness and to assess whether we received the correct dataset after which two studies were excluded (SupMat2). Next, data were harmonized (see the coding of subgroups or harmonization of outcome measures) and merged. SupMat4 provides an overview of included studies. Combined, these studies included 39,793 primary, middle, or high school participants aged five to 20 years (*M*_age_ = 12.58, *SD* = 2.34). All study procedures were approved by the TNO research ethics board (ethics committee file number: 2019–85).

### Coding of Subgroups (“Whom”)

Subgroups identified in our analyses pertained to age, sex, ethnicity, SES, and initial severity of bullying and victimization. Sex was coded as 0 = *boys* and 1 = *girls*. We differentiated between younger (< age 12) and older participants. SES was defined as low, medium, or high. If this estimation was not already made within studies, we estimated the within-study SES variability and coded values 1.5 *SD* below mean as low SES, values 1.5 *SD* above mean as high SES, and the rest as medium SES. Ethnicity was coded as minority or non-minority. If not already included in the dataset, we coded ethnicity as non-minority if participants were born in the study country of origin or if their native language was that of the study country. Baseline levels of bullying perpetration and victimization were used as indicators of problem severity before intervention.

### Coding of Intervention Components (“What”)

MH and BM coded intervention components based on information provided in papers and supplementary materials (SupMat4). Disagreements were discussed and adjusted accordingly. PIs were consulted to verify the coding. We used a coding scheme based on previous schemes by Farrington and Ttofi (2009), Gaffney et al. (2019), and De Mooij et al. (2020a; SupMat3). We coded whether the intervention included a school anti-bullying policy, school assemblies (during which students were informed about bullying or collective psychoeducation), playground supervision (including an increase in supervision in hotspots), a monitor which identifies bullies, victims, and possible other bullying roles and reports back to school personnel, classroom rules, classroom placement strategies (changing seating arrangements to prevent bullying or to intervene after a bullying incident), peer involvement, and disciplinary methods. Disciplinary methods could be punitive (focusing on confronting the bully and insisting on changing behavior) or non-punitive (focusing on a positive approach, e.g., increasing empathy for victims).

Child-focused components of interventions were also coded. We coded whether the intervention included psychoeducation (transferring knowledge about bullying and/or victimization), psychophysical exercises (focused on physical relaxation, assertiveness, and resilience), interpersonal skill-building (exercises to improve prosocial or [non]verbal communication skills), or cognitive-emotional skill-building (intrapersonal skills aimed at improving the recognition and adequate regulation of emotions and thoughts). The interventions only included psychoeducation and cognitive-emotional skill-building components but not psychophysical exercises and interpersonal skill-building, which were thus omitted from our analyses. Because all interventions included psychoeducation, we could not compare interventions with and without psychoeducation. Additionally, only one intervention included peer involvement and students’ active engagement, and thus this component was not included in our analyses.

### Harmonization of Bullying and Victimization Outcome Measures

All studies measured bullying perpetration and victimization with (an adapted version of) the Olweus bullying and victimization questionnaire (Olweus, 1996), which uses a frequency scale; participants indicated how often they bullied and were victimized by others. Some studies used one general question to assess bullying and victimization (“How often were you bullied/did you bully in the past/this term”) that was answered on a 5-point scale. Others used a multiitem questionnaire that taps into specific forms of bullying (e.g., kicking and hitting, gossiping, vandalizing other’s property), resulting in a sum score. We harmonized the different outcome measures into one outcome measure (SupMat5), and created a clinically relevant dichotomized outcome measure by combining categories “never and rarely bullied/bully” and categories “regularly and daily bullied/bully.”

### Risk of (Publication) Bias

Risk of bias in the included studies was assessed on their bias in participant selection, classification of interventions, deviations from the intended intervention, missing data, and measurement of outcomes (Sterne et al., 2016). This provided an overall bias score (low, moderate, or serious) per study (SupMat6). Bias was assessed by BM and SK (ICC = 0.72). None of the studies had a serious risk of bias score. We also assessed whether included studies differed from eligible studies that were not included in our IPD. We found no significant differences based on the year of publication, location and design of the study, and reported effects (SupMat6).

### Statistical Analyses

A one-stage meta-analysis with random intercepts at the study level was conducted on the pooled dataset of harmonized study data. Participants (level 1) were nested in schools (level 2), and nested in intervention studies (level 3), which was accounted for by fitting multilevel regression models. Most datasets did not include a variable identifying what school participants were in (i.e., they only coded whether participants were in the intervention or control condition), so we fitted two-level regression models to correct for variance explained at the study level. We estimated logistic regression models using odds ratios, 95% confidence intervals, and -2 log-likelihood [-2LL] fit estimates. We treated missing data in our univariate analyses with listwise deletion; this was done for computational efficiency given our large dataset and focused analyses. We conducted separate univariate regression analyses for the postintervention outcomes of victimization and bullying perpetration. The entire pooled dataset (*n* = 39,793) was used for our primary analyses and to assess *for whom* the interventions work best. We created a subgroup (*n* = 22,101) by omitting all participants that did not receive an intervention to assess *what works* in school-based anti-bullying interventions and *what works for whom.*

To control type I error rate, we applied a Benjamini–Hochberg FDR correction (25%). Critical levels of interaction effects were corrected per subgroup analysis. Effect sizes for odds ratios were calculated using Hasselblad and Hedges’ method (1995). Additionally, “leave-one-out” sensitivity analyses were conducted to assess the stability of significant findings to further guard against type I errors (SupMat10). Analyses were repeated, excluding one study at a time, to assess if specific studies drove results. In line with the data-sharing agreement, studies that changed the results were not disclosed.

## Results

In the total sample, 4698 participants (16.1%) reported being victimized regularly to daily, and 2142 participants (7.6%) reported bullying at least regularly to daily. To compare participants from different subgroups on postvictimization and perpetration levels, we conducted univariate multilevel logistic regression analyses. We controlled for variance explained at the level of study characteristics. Results indicated that participants who were victimized at baseline were more likely to be victimized at posttest (OR = 6.149, *p* <.001), and participants who bullied at baseline were more likely to bully at posttest (OR = 8.480, *p* < .001). Girls were less likely to be victimized or to bully at posttest (victimization: OR = 0.772, *p* < .001, perpetration: OR = 0.443, *p* < .001). Older participants were less likely to be victimized at posttest (OR = 0.888, *p* < .001) yet were more likely to bully at posttest (OR = 1.089, *p* < .01). Participants from ethnic minorities were more likely to be victimized and to bully at posttest (victimization: OR = 1.222, *p* < .01, perpetration: OR = 1.380, *p* < .01). And participants from high and medium SES were less likely to be victimized at posttest (high SES: OR = 0.560, *p* < .001, medium SES: OR = 0.745, *p* = .018); no differences were found for posttest perpetration. See SupMat7 for more test statistics and baseline comparisons of subgroups. Pearson’s correlation between self-reported bullying and victimization was *r* = 0.23 (*p* < .001); participants who were victimized more often also reported bullying more.

### Do School-Based Anti-bullying Interventions Work?

The two univariate, multilevel logistic regressions demonstrated that school-based anti-bullying interventions significantly reduced victimization (*t* =  − 6.61, OR = 0.77, *95% CI* = 0.71; 0.83, *p* < .001, *d* =  − 0.14) and bullying perpetration (*t* =  − 2.30, OR = 0.88, *95% CI* = 0.79; 0.98, *p* < .05, *d* =  − 0.07) in schools. Leave-one-out sensitivity analyses indicated that effects were unaffected by exclusion of all (victimization) or almost all (*n*-2) studies (bullying perpetration).

### For Whom Do School-Based Anti-bullying Interventions Work?

Results of the multilevel logistic regression models found no significant differential effects in reducing victimization across different subgroups (of sex, age, ethnicity, SES, and initial bullying or victimization levels). Across almost all subgroups, results indicated no differential reductions in reported victimization (Table 1, SupMat8). There was one exception: anti-bullying interventions were more effective in reducing victimization in participants who reported higher initial victimization before the intervention compared with participants who reported lower initial victimization. Sensitivity analyses showed that this result was affected by the exclusion of four individual studies. Results also indicated that no differential reductions were found in reported perpetration across almost all subgroups, with one exception: anti-bullying interventions reduced bullying perpetration more in younger (< 12 years) than in older participants. Sensitivity analyses indicated that this result was unaffected by exclusion of almost all (*n*-1) studies.

**Table 1:** Interaction effects of subgroup × intervention status on postintervention victimization and bullying perpetration (for whom does it work)

**Victimization model**	Coefficient	*SE*	*t*	Sig	Exp. (coefficient)	95% CI (coef.)	Rank	Adj. *a*
						[LL, UL]		
Sex	− 0.262	0.057	− 4.599	< .001	0.769	[0.69, 0.86]		
Intervention	− 0.305	0.054	− 5.679	< .001	0.737	[0.66, 0.82]		
Sex * intervention	0.093	0.078	1.197	.231	1.098	[0.94, 1.28]	4	.167
Age	− 0.158	0.089	− 1.768	.077	0.854	[0.72, 1.02]		
Intervention	− 0.306	0.058	− 5.286	< .001	0.736	[0.66, 0.83]		
Age * intervention	0.175	0.091	1.919	.055	1.191	[0.99, 1.42]	2	.083
Ethnicity	0.286	0.120	2.384	.017	1.330	[1.05, 1.68]		
Intervention	− 0.277	0.044	− 6.368	< .001	0.758	[0.70, 0.83]		
Ethnicity * intervention	− 0.141	0.151	− 0.933	.351	0.868	[0.65, 1.17]	6	.250
SES high	− 0.614	0.191	− 3.221	.001	0.541	[0.37, 0.79]		
SES medium	− 0.386	0.181	− 2.129	.033	0.680	[0.48, 0.97]		
Intervention	− 0.282	0.216	− 1.307	.191	0.754	[0.49, 1.15]		
SES (high) * intervention	0.390	0.276	1.414	.157	1.477	[0.86, 2.54]	3	.125
SES (medium) * intervention	0.307	0.259	1.186	.236	1.360	[0.82, 2.26]	5	.208
Initial victimization	1.913	0.061	31.398	< .001	6.775	[6.01, 7.64]		
Intervention	− 0.202	0.049	− 4.145	< .001	0.817	[0.74, 0.90]		
Initialvictimiz.* intervention	− 0.168	0.081	− 2.065	.039	0.845	[0.72, 0.99]	1	.042
**Perpetration model**	Coefficient	*SE*	*t*	Sig	Exp (coefficient)	95% CI (coef.)	Rank	Adj. *a*
						[LL, UL]		
Sex	− 0.630	0.082	− 7.684	< .001	0.533	[0.45, 0.63]		
Intervention	− 0.105	0.068	− 1.540	.124	0.901	[0.79, 1.03]		
Sex * intervention	− 0.068	0.113	− 0.601	.548	0.934	[0.75, 1.17]	5	.208
Age	− 0.022	0.115	− 0.191	.848	0.978	[0.78, 1.23]		
Intervention	− 0.342	0.091	− 3.756	< .001	0.710	[0.59, 0.85]		
Age * intervention	0.357	0.121	2.954	.003	1.429	[1.13, 1.81]	1	.042
Ethnicity	0.112	0.166	0.675	.500	1.119	[0.81, 1.55]		
Intervention	− 0.148	0.059	− 2.514	.012	0.863	[0.77, 0.97]		
Ethnicity * intervention	0.252	0.208	1.212	.226	1.287	[0.86, 1.94]	2	.083
SES high	− 0.132	0.265	− 0.498	.619	0.877	[0.52, 1.47]		
SES medium	− 0.200	0.276	− 0.723	.470	0.819	[0.48, 1.41]		
Intervention	0.062	0.335	0.186	.852	1.064	[0.55, 2.05]		
SES (high) * intervention	0.349	0.379	0.920	.358	1.417	[0.67, 2.98]	3	.125
SES (medium) * intervention	0.266	0.399	0.666	.506	1.304	[0.60, 2.85]	4	.167
Initial perpetration	2.160	0.089	24.354	< .001	8.670	[7.29, 10.32]		
Intervention	− 0.115	0.064	− 1.803	.071	0.891	[0.79, 1.01]		
Initialperpetr * intervention	− 0.042	0.121	− 0.347	.728	0.959	[0.76, 1.22]	6	.250

### What Works for Whom in School-Based Anti-bullying Interventions?

Multilevel logistic regression models found no evidence supporting that interventions worked differently depending on the use of specific intervention components (Table 2, SupMat8). There was one exception: interventions including non-punitive disciplinary methods had an iatrogenic effect on bullying perpetration and victimization compared with interventions that did not use any disciplinary methods. Sensitivity analyses showed this effect was affected by the exclusion of four individual studies.

**Table 2:** Exploration of main effects of intervention components on postintervention victimization and bullying perpetration (what works)

**Victimization model**	Coefficient	*SE*	*t*	Sig	Exp (coefficient)	95% CI (coef.)	Rank	Adj. *a*
						[LL, UL]		
School policy	0.638	0.369	1.727	.084	1.892	[0.92, 3.90]	3	.094
Monitor	0.097	0.551	0.176	.861	1.102	[0.37, 3.25]	8	.250
Classroom rules	0.638	0.369	1.727	.084	1.892	[0.92, 3.90]	2	.063
School assemblies	0.077	0.363	0.211	.833	1.080	[0.53, 2.20]	6	.186
Playground supervision	0.077	0.363	0.211	.833	1.080	[0.53, 2.20]	7	.219
Disciplinary methods								
*Non-punitive*	0.879	0.402	2.187	.029	2.408	[1.10, 5.29]	1	.031
*Non-punitive and punitive*	0.466	0.381	1.223	.221	1.594	[0.76, 3.37]	5	.156
Cognitive-emotional	1.049	0.697	1.504	.132	2.854	[0.73, 11.19]	4	.125
**Perpetration model**	Coefficient	*SE*	*t*	Sig	Exp (coefficient)	95% CI (coef.)	Rank	Adj. *a*
						[LL, UL]		
School policy	1.270	0.723	1.758	.079	3.561	[0.86, 14.68]	3	.107
Monitor	− 0.293	0.955	− 0.306	.759	0.746	[0.12, 4.85]	5	.179
Classroom rules	1.270	0.723	1.758	.079	3.561	[0.86, 14.68]	2	.071
School assemblies	0.118	0.675	0.175	.861	1.125	[0.30, 4.23]	7	.250
Playground supervision	0.118	0.675	0.175	.861	1.125	[0.30, 4.23]	6	.214
Disciplinary methods								
*Non-punitive*	1.782	0.776	2.297	.022	5.940	[1.30, 27.16]	1	.036
*Non-punitive and punitive*	0.894	0.690	1.296	.195	2.444	[0.63, 9.44]	4	.143

Next, we tested whether intervention components had differential effects across subgroups (SupMat9). For some combinations, not enough participants were available in each cell (e.g., ethnicity and cognitive-emotional skill-building combined), so these were removed from analyses. We did not find significant interaction effects for victimization and perpetration between individual intervention components and age, ethnicity, and SES subgroups. For sex, however, analyses did show that interventions that included non-punitive disciplinary methods had iatrogenic effects on victimization levels that were stronger for girls than for boys. Sensitivity analyses showed that this interaction effect was unaffected by the exclusion of almost all (*n*-2) studies. The what works for whom analyses also showed that school assemblies and playground supervision had iatrogenic effects on perpetration for participants who bullied regularly to daily at baseline, compared with participants who never or rarely bullied at baseline. The sensitivity analyses showed that this result was unaffected by the exclusion of almost all (*n-1*) studies.

## Discussion

This individual participant data (IPD) meta-analysis assessed the intervention effects of school-based anti-bullying interventions among 39,793 children and adolescents and found that anti-bullying interventions effectively reduce victimization and bullying perpetration. Contrary to our expectations, we could not find evidence indicating that anti-bullying interventions work differently for girls and boys, ethnic minorities and majorities, youth with low, middle, and high SES, and youth with low and high initial perpetration levels. There were two exceptions: children below the age of 12 benefited more from anti-bullying interventions than older adolescents, and youth with high initial victimization levels benefited more from anti-bullying interventions than youth with low initial victimization levels before the intervention. In addition, we found no evidence indicating that intervention effects depended on the inclusion of specific intervention components, except for interventions that contained non-punitive disciplinary methods, which yielded iatrogenic effects on bullying perpetration and victimization. Additionally, we found that these iatrogenic intervention effects of non-punitive disciplinary methods were stronger for girls’ victimization levels and that school assemblies and playground supervision had iatrogenic effects on bullying perpetration for youth who bullied regularly to daily at baseline.

### School-Based Anti-bullying Intervention Effects

Our findings show that school-based anti-bullying interventions yield favorable effects in reducing bullying and victimization, which is in line with previous meta-analyses (De Mooij et al., 2020a; Gaffney et al., 2019a, b; Ttofi & Farrington, 2009). The effects seem to be statistically small. Perhaps, because school-wide anti-bullying interventions target all children and adolescents in the school, even youth who are not victimized or who do not bully. These small effects are consistent with the effects of other whole-school programs with a universal approach (Greenberg & Abenavoli, 2017). Despite small effects, the clinical importance of the decrease in bullying is high. Victims and bullies are at risk for maladjustment; even when only small groups of victimized or bullying youth benefit, this is critical for their healthy development (Ttofi et al., 2016).

### For Whom Do School-Based Anti-bullying Interventions Work

Looking at subgroups of children and adolescents, this IPD meta-analysis could not find evidence supporting our hypothesis that school-based anti-bullying interventions work differently for boys and girls, youth from ethnic majority and minority groups, youth with different levels of initial perpetration, and youth from different SES backgrounds. This may indicate that anti-bullying programs are effective across many contexts and populations. However, two interesting differences emerged. First, anti-bullying interventions were more effective for youth with high initial levels of victimization than those with low initial levels of victimization. This finding, which was in line with previous research outcomes (Ferguson et al., 2007; Juvonen et al., 2016–but see Kaufman et al. (2018) for an exception), suggests that interventions implemented school-wide may successfully target youth that need it most. This finding may be explained by the simple fact that there is more room for behavioral improvement in youth who report more initial victimization. These children and adolescents may have a higher motivation to engage with the intervention or a higher likelihood to be targeted by some intervention components. Notably, as bullying perpetration also decreased in general, severely victimized youth may benefit most from the general decrease in perpetration because they are most confronted with bullying.

The second finding that emerged, which was more robust, was that school-based anti-bullying interventions were less effective in reducing bullying perpetration for adolescents of 12 years and older. This finding aligns with our hypothesis (Yeager et al., 2015). Perhaps a disconnect between current anti-bullying intervention approaches and the changing nature of bullying in adolescence causes these differential effects for age. Specifically, anti-bullying interventions implemented in adolescence need a different “tone” with a stronger emphasis on adolescents’ strive for autonomy and respect (Yeager et al., 2018). Adolescents may be more sensitive than younger children to being treated with respect and less willing to accept the authority of adults (Yeager et al., 2018). Also, the motivation to bully might differ between adolescents and children, with adolescents focusing more on gaining status by bullying (Volk et al., 2012). Thus, one interesting possibility may be to gear anti-bullying interventions in adolescence towards offering prosocial ways to gain popularity (Yeager et al., 2018). Also, because bully-victim patterns may have become more fixed in adolescence (e.g., Sentse et al., 2015), interventions aimed at adolescents may need to be more intensive, targeting those directly involved in bullying perpetration to change their mindset (Yeager et al., 2018).

### What Might (Not) Work for Whom?

Although explorative, our findings indicate that there might be subgroup differences in what works in school-based anti-bullying interventions. For example, non-punitive disciplinary methods seemed to yield iatrogenic effects in general, but with even higher postintervention levels of victimization for girls. However, because of the explorative nature of this analysis and the lack of robustness of this particular finding, future studies should investigate this further. Another, more robust, finding indicated that, compared with interventions that did not include these components, both school assemblies and playground supervision may yield iatrogenic effects in children and adolescents with high initial levels of perpetration. Compared with youth with low initial levels of perpetrations, these children and adolescents reported higher instead of lower levels of perpetration after interventions that included school assemblies and playground supervision.

How can we explain such effects? Both components are geared towards increasing the visibility of perpetration, and publicly addressing this issue, which might teach youth new ways of bullying others. Moreover, publicly addressing bullying might make the initial bully feel exposed or told on, which might increase anger and a desire for revenge. Yet other explanations could be that in publicly addressing bullying, teachers are inadvertently enhancing the bully’s image and reputation of being in power, or that this public address leads to an increased awareness of what bullying is, leading bullies to report more bullying perpetration than before. Addressing the school as a whole and publicly condemning and punishing bullying, as is done with these components within universal programs, comes with the risk of reaching subgroups of youths who react differently to these measures. School assemblies and playground supervision may work well as preventive measures for youth who do not often bully others but may be counterproductive for a subgroup of more severe bullies.

### Strengths and Limitations

Our IPD meta-analysis assessed the effects of school-based anti-bullying interventions using an innovative approach, yielding superior power, based on a large sample size of 39,793 children and adolescents. Collecting data from different individual studies allowed us to build a comprehensive dataset on school-based anti-bullying intervention effects and to perform a novel investigation focused on for whom anti-bullying interventions work as well as to explore which specific anti-bullying intervention components might work (or yield iatrogenic effects) for subgroups of youth.

Several limitations of our approach also warrant mentioning. First, we did not consider implementation level and quality. The determination of intervention components was based on the presence of these components in the manuals and not on the actual implementation of the intervention components during the studies. This may limit the generalizability of our findings, given that a certain implementation threshold or intervention dosage may be a prerequisite for establishing intervention effects in the first place (De Mooij et al., 2020a; Horner et al., 2006). It is important to include the level of implementation of the intervention (components) in studies on the effectiveness of anti-bullying interventions. Another limitation is that we did not investigate longer-term intervention effects. The sustainability of both the implementation and effects may be different across specific components. Also, our findings should be interpreted carefully because we based our analysis on interventions that combine different components. We cannot exclude the possibility that our effect estimates for specific components are to some extent dependent on the specific combination with other components, yielding synergistic, or 1 + 1 = 3, effects (Low & van Ryzin, 2014). Furthermore, due to harmonization purposes, we were limited in operationalizing ethnicity and SES. Ethnic minorities and majorities might be identified differently in different countries. We calculated high, medium, or low SES within studies making it harder to assess the relative SES between studies. This led to quite some variation in SES within two out of four studies. However, the other two studies had some underrepresentation of either low SES (3.1%) or high SES (0%). Findings should be interpreted with these limitations in mind.

Finally, this IPD meta-analysis does not provide a comprehensive representation of the entire body of anti-bullying intervention research but rather represents a non-representative set of studies (published in peer-reviewed scientific journals) for which analyzable IPD could be obtained. The findings should be generalized with caution outside of Europe, as the continents Asia and Africa are not represented and the continents America and Oceania both only account for 10% of the included studies. The manifestation of bullying may differ between countries, which could reflect social and cultural differences in bullying and might also have different implications for national policies (e.g., Craig et al., 2009). In addition, our sensitivity analyses suggest that although most findings came out generally robust, some findings might have been statistically more dependent on the in- or exclusion of specific studies. Lastly, our study would benefit from more heterogeneity in the included intervention programs; currently, one intervention program is represented largely (i.e., KiVa) while others are missing. Our future aim is to replicate these findings in an IPD meta-analysis with more studies, allowing for an even more stringent analysis of a more heterogeneous set of intervention programs and components implemented worldwide.

### Future Research Directions

In general, future school-based anti-bullying intervention research could benefit from examining program effects in different subgroups instead of only assessing effects in the sample as a whole. In addition, it may be worthwhile to examine specific components instead of complete packages. Possibly, not all interventions can be implemented harmlessly for everyone; caution is warranted. To further explore the “what works for whom” question, we encourage future scholars to use experimental designs to sort out component effects for specific subgroups of youths, such as factorial designs (Bonsergent et al., 2013) or microtrials (De Mooij et al., 2020b, c). Another interesting new approach in testing component effectiveness is to assess clusters of components together with network meta-analyses (Cartose et al., 2019). Future studies can further differentiate between the degree of victimization and bullying; how do (components of) interventions affect youths who are severely victimized or who severely bully others before interventions? Although beyond the scope of our study, previous studies indicated that interventions might work less well or have iatrogenic effects on other developmental outcomes for these children and adolescents, which is worthy of further investigation (e.g., Huitsing et al., 2019; Kaufman et al., 2018).

### Implications and Conclusion

School-based anti-bullying interventions generally reduce bullying victimization and perpetration. Our findings do not provide evidence that interventions were differentially effective across different subgroups of sex, ethnic background, and SES, but anti-bullying interventions implemented to reduce perpetration among adolescents above 12 are less effective. This study shows that anti-bullying interventions are more effective in reducing victimization for youth with higher initial victimization levels. Although this is generally promising, it also suggests that we may need more preventive strategies to help youth who are only “sometimes” victimized. Further research is needed to answer the “what works for whom” question, with appropriate research designs that allow for disentangling specific component effects.

Our findings highlight the importance of tailoring interventions to children and adolescents’ age and initial victimization levels. Tailoring interventions enables maximizing intervention efforts (where effect sizes now seem relatively small). However, practical implications might be challenging. Although tailoring interventions for individual students seems promising, we cannot target group norms or peer group dynamics as effectively as whole-school programs. The solution might be somewhere in the middle: going beyond the single whole-school program towards a multitier approach of interventions. Interventions should have the ability to be flexibly deployed by using both universal, selected, and indicated components. Moving away from a “one size fits all” to a multitier approach enables schools to effectively meet the needs of the different subgroups that comprise the school populations while also addressing the norms and group dynamics of the entire school population.

### Supplementary Information

Below is the link to the electronic supplementary material.

Supplementary file1 (DOCX 654 KB)

Supplementary file2 (DOCX 24 KB)

## Data Availability

The dataset is in development, data are currently not available for sharing.
