# Supplementary material for: What Works for Whom in School-Based Anti-bullying Interventions? An Individual Participant Data Meta-analysis
Source: Prev Sci. 2022 Jul 7;24(8):1435–46. doi: 10.1007/s11121-022-01387-z (PMC10678813; doi:10.1007/s11121-022-01387-z)
Supplement: Supplementary file 1 — Supplementary file1 (DOCX 654 KB) [file 11121_2022_1387_MOESM1_ESM.docx]

**Supplementary Materials**

**Title:** What Works for Whom in School-Based Anti-Bullying Interventions? An Individual Participant Data Meta-Analysis

**Journal:** Special Issue: Innovations and Applications of Integrative Data Analysis (IDA) and Related Data Harmonization Procedures in Prevention Science

**Authors:** Maud Hensums, Brechtje de Mooij, Steven C. Kuijper, BIRC*, Minne Fekkes, & Geertjan Overbeek

*BIRC: the anti-**B**ullying **I**nterventions **R**esearch **C**onsortium – participating PIs (and co-authors of this manuscript) are, in alphabetical order: Donna Cross, Ann DeSmet, Claire F. Garandeau, Katja Joronen, Bonnie Leadbeater, Ersilia Menesini, Benedetta Emanuela Palladino, Christina Salmivalli, Olga Solomontos-Kountouri, and René Veenstra

**Corresponding Author:** Correspondence concerning this article should be addressed to Maud Hensums, University of Amsterdam, Nieuwe Achtergracht 127, 1018WS Amsterdam. Email: [M.Hensums@uva.nl](mailto:M.Hensums@uva.nl)

**S1.** Search Strings for PsychINFO (search strings are adjusted accordingly to other databases)

**Full Search String for PsychINFO**

#1 (cyber)bullying and/or (cyber)victimization

bullying/ OR cyberbullying/ OR (bullies OR bully* OR victimi*ation OR victimi*ed OR peer harassment* OR cyberbull* OR cybervictimi*).ti,ab,id.

#2 Intervention

intervention/ OR training/ OR school based intervention/ OR group intervention/ OR curriculum/ OR (training* OR intervention* OR program*).ti,ab,id.

#3 children and adolescents (6-18 years old)

(school age 6 12 yrs OR adolescence 13 17 yrs).ag. OR elementary school students/ OR primary school students/ OR middle school students/ OR junior high school students/ OR high school students/ OR (child* OR kid OR kids OR prepubescen* OR prepuberty* OR teen* OR young* OR youth* OR juvenile* OR girl* OR boy* OR preadolesc* OR adolesc* OR elementary school* OR primary school* OR K-12* OR K12 OR 1st-grade* OR first-grade* OR grade 1 OR grade one OR 2nd-grade* OR second-grade* OR grade 2 OR grade two OR 3rd-grade* OR third-grade* OR grade 3 OR grade three OR 4th-grade* OR fourth-grade* OR grade 4 OR grade four OR 5th-grade* OR fifth-grade* OR grade 5 OR grade five OR 6th-grade* OR sixth-grade* OR grade 6 OR grade six OR intermediate general OR secondary education OR secondary school* OR 7th-grade* OR seventh-grade* OR grade 7 OR grade seven OR 8th-grade* OR eight-grade* OR grade 8 OR grade eight OR 9th-grade* OR ninth-grade* OR grade 9 OR grade nine OR 10th-grade* OR tenth-grade* OR grade 10 OR grade ten OR 11th-grade* OR eleventh-grade* OR grade 11 OR grade eleven OR 12th-grade* OR twelfth-grade* OR grade 12 OR grade twelve OR junior high* OR highschool*).ti,ab,id.

#4 Study type

(followup study OR "treatment outcome/clinical trial").md. OR followup studies/ OR (random* OR longitud* OR ((follow up OR followup) ADJ3 (study OR studies)) OR ((interaction OR direct OR indirect OR causal OR generali#ed OR treatment) ADJ1 (effect OR effects)) OR (control ADJ3 group*) OR repeated measure* OR treatment condition* OR control condition* OR quasi experiment* OR quasiexperiment* OR RCT).ti,ab,id.

**S2. Flowchart for Inclusion of Studies in IPD Meta-Analysis.**

**
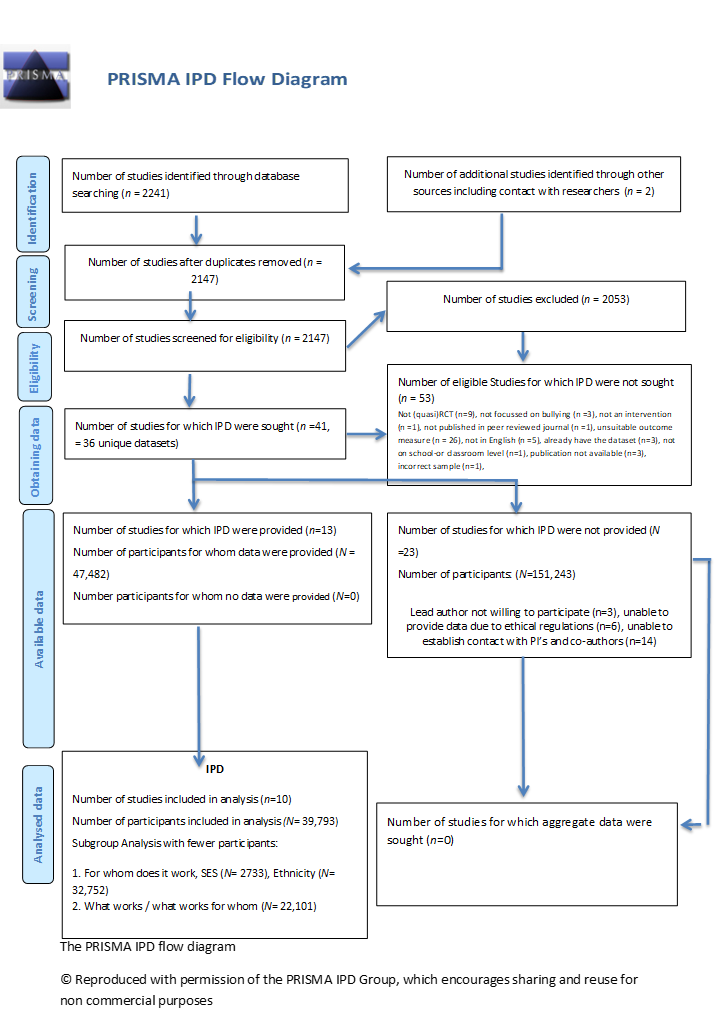
**

**S3.** Coding Scheme Intervention Components

**Coding scheme IPD anti-bullying programs**

Bullying Interventions Research Consortium (BIRC)

**Information coders:**

Name coder: …………………………………………………………………………………………...

Date of coding: …………………………………………………………………………………………...

Name second coder:…………………………………………………………………………………………...

Date discussion between coders: …………………………………………………………………………………………...

**Information article:**

First author:…………………………………………………………………………………………...

Title of article (in short):…………………………………………………………………………………………...

**Information program:**

Name of the program: …………………………………………………………………………………………...

Information from Manual

or article? …………………………………………………………………………………………...

**References:**

De Mooij, B., Fekkes, M., Scholte, R.H.J., & Overbeek, G. (2020). Effective components of social skills training programs for children and adolescents in nonclinical samples: A multilevel meta-analysis. *Clinical Child and Family Psychology Review,* 1–15. https://doi.org/10.1007/s10567-019-00308-x

Farrington, D. P., & Ttofi, M. M. (2009). School-based programs to reduce bullying and victimization. *The Campbell Collaboration*, *6*, 1-149.

| - - - 1. PROGRAM COMPONENTS | |
| --- | --- |
| 1. Theory of change   *Which mechanisms of change does the program target (what is most important).*  *Open question.* | …………………………………………………  ❑ Unclear (999) |
| 1. Definition of bullying   *How is bullying defined by the program.*  *(Olweus: repetitive, intentional, power inbalance).* | ❑ Based on Olweus (0)  ❑ Other (1)  ❑ Unclear (999) |
| 1. School anti bullying policy   *Presence of a formal anti-bullying policy on behalf of the school* | ❑ No (0)  ❑ Yes (1)  ❑ Unclear (999) |
| 1. Monitor   *Does the program use a bully/victim monitor to identify and address students’ roles.* | ❑ No (0)  ❑ Yes (1)  ❑ Unclear (999) |
| 1. Classroom rules   *The use of rules against bullying that*  *students are expected to follow* | ❑ No (0)  ❑ Yes (1)  ❑ Unclear (999) |
| 1. School assemblies   *School assemblies during which children were informed about bullying (collective psychoeducation)* | ❑ No (0)  ❑ Yes (1)  ❑ Unclear (999) |
| 1. Student placement   *Are teachers instructed to change the seating arrangements to prevent bullying or to intervene after a bullying incident* | ❑ No (0)  ❑ Yes (1)  ❑ Unclear (999) |
| 1. Work with peers   *Formal engagement of peers in tackling bullying (e.g. mediation, peer mentoring).* | ❑ No (0)  ❑ Yes (1)  ❑ Unclear (999) |
| 1. Improved playground supervision   *Identification of hotspots/hot-times for bullying and increasing supervision.* | ❑ No (0)  ❑ Yes (1)  ❑ Unclear (999) |
| 1. Disciplinary methods   *Use of punitive methods in dealing with bullying situations (e.g. expelling bully).*  *Use of non-punitive methods in dealing with bullying situations (e.g. restoring the harm that has been done, “positive” approach).* | ❑ None (0)  ❑ Punitive methods (1)  ❑ Non-punitive methods (2)  ❑ Punitive and non-punitive methods (3)  ❑ Unclear (999) |

| - - - 1. CHILD-FOCUSED TRAINING - CONTENT | |
| --- | --- |
| 1. Psychoeducation   *Children are informed about bullying, changing attitudes* | ❑ Not included (0)  ❑ Included (1)  ❑ Unclear (999) |
| 1. Psychophysical   *Relaxation, posture, etc.* | ❑ Not included (0)  ❑ Included (1)  ❑ Unclear (999) |
| 1. Social skills   *(Non-)verbal communication skills, engagement, intepersonal problem solving skills, etc.* | ❑ Not included (0)  ❑ Included (1)  ❑ Unclear (999) |
| 1. Cognitive-emotion skills   *Emotion recognition (own or other’s), impulse regulation, cognitive restructuring (transforming unhelpful thoughts into helpful thoughts), empathy (understanding other’s behavior)* | ❑ Not included (0)  ❑ Included (1)  ❑ Unclear (999) |

**S4. Included Trials and Trial Information**

Cross, D., Monks, H., Hall, M., Shaw, T., Pintabona, Y., Erceg, E., Hamilton, G., Roberts, C., Waters, S., & Lester, L. (2011). Three-year results of the Friendly Schools whole-of-school intervention on children’s bullying behaviour. *British Educational Research Journal, 37*, 105–129. <https://doi.org/10.1080/01411920903420024>

DeSmet, A., Bastiaensens, S., Van Cleemput, K., Poels, K., Vandebosch, H., Deboutte, G., Herrewijn, L., Malliet, S., Pabian, S., Van Broeckhoven, F., De Troyer, O., Deglorie, G., Van Hoecke, S., Samyn, K., & Bourdeaudhuij, I. (2018). The efficacy of the Friendly Attack serious digital game to promote prosocial bystander behavior in cyberbullying among young adolescents: A cluster-randomized controlled trial. *Computers in Human Behavior, 78*, 336*─*347. <https://doi.org/10.1016/j.chb.2017.10.011>

Huitsing, G., Lodder, G.M.A., Browne, W.J., Oldenburg, B., Van der Ploeg, R., Veenstra, R. (2020). A large-scale replication of the effectiveness of the KiVa antibullying program: A randomized controlled trial in the Netherlands. *Prevention Science, 21*, 627*─*638. <https://doi.org/10.1007/s11121-020-01116-4>

Joronen, K., Konu, A., Rankin, H. S., & Åstedt-Kurki, P. (2011). An evaluation of a drama program to enhance social relationships and anti-bullying at elementary school: A controlled study. *Health Promotion International, 27*, 5–14. <https://doi.org/10.1093/heapro/dar012>

^[[1]](#footnote-2)^Juvonen, J., & Schacter, H. L. (2016). “Can a school-wide bullying prevention program improve the plight of victims? Evidence for risk × intervention effects”: Correction to Juvonen et al. (2016). *Journal of Consulting and Clinical Psychology, 84*(6), 483–483. <https://doi.org/10.1037/ccp0000116>

Kärnä, A., Voeten, M., Little, T. D., Poskiparta, E., Kaljonen, A., & Salmivalli, C. (2011). A large‐scale evaluation of the KiVa antibullying program: Grades 4–6. *Child development, 82*(1), 311−330. <https://doi.org/10.1111/j.1467-8624.2010.01557.x>

Kärnä, A., Voeten, M., Little, T. D., Alanen, E., Poskiparta, E., & Salmivalli, C. (2013). Effectiveness of the KiVa Antibullying Program: Grades 1–3 and 7–9. *Journal of Educational Psychology, 105*(2), 535. doi:10.1037/a0030417

Leadbeater, B., & Sukhawathanakul, P. (2011). Multicomponent programs for reducing peer victimization in early elementary school: A longitudinal evaluation of the WITS primary program. *Journal of Community Psychology, 39*(5), 606-620. <https://doi.org/10.1002/jcop.20447>

Nocentini, A., & Menesini, E. (2016). KiVa anti-bullying program in Italy: Evidence of effectiveness in a randomized controlled trial. *Prevention Science, 17*, 1012–1023. <https://doi.org/10.1007/s11121-016-0690-z>

Palladino, B.E., Nocentini, A., & Menesini, E. (2016). Evidence-based intervention against bullying and cyberbullying: Evaluation of the NoTrap! Program in two independent trials. *Aggressive Behavior, 42*, 194–206. doi:10.1002/ab.21636

Salmivalli, C., Kaukiainen, A., & Voeten, M. (2005). Anti-bullying intervention: Implementation and outcome. *British Journal of Educational Psychology, 75*(3), 465–487. https://doi.org/http://dx.doi.org/10.1348/000709905X26011

Solomontos-Kountouri, O., Gradinger, P., Yanagida, T., & Strohmeier, D. (2016). The implementation and evaluation of the ViSC program in Cyprus: Challenges of cross-national dissemination and evaluation results. *European Journal of Developmental Psychology, 13*(6), 737–755. <https://doi.org/10.1080/17405629.2015.1136618>

**Table 1**

*Study and Trial Characteristics.*

| **Trial** | **Intervention** | **Country** | ***N*** | **Setting** | **Age (Mean, *SD*)** | | | **Sex (*n*)** | | **SES (%)** | | **Risk of Bias** |
| --- | --- | --- | --- | --- | --- | --- | --- | --- | --- | --- | --- | --- |
| Solomontos-Kountouri et al. (2016) | ViSC | Cyprus | 1652 | Middle school | 12.62 | 0.61 |  | Girls | 801 | Low  Middle | 10.9 30.3 | Moderate |
| DeSmet et al. (2018) | Friendly Attac | Belgium | 251 | Middle school | 13.99 | 0.68 |  | Girls | 143 | Low  Middle | 2.0 25.1 | Moderate |
| Joronen et al. (2011) | Drama Program | Finland | 134 | Primary school | 10.39 | 0.67 |  | Girls | 67 | Low  Middle | NA NA | Moderate |
| Leadbeater et al. (2012) | WITS | Canada | 830 | Primary school | 7.33 | 0.85 |  | Girls | 411 | Low  Middle | 21.3 77.2 | Moderate |
| Nocentini & Menesini (2016) | KiVa (Italy) | Italy | 2184 | Primary and Middle school | 9.92 | 1.14 |  | Girls | 1001 | Low  Middle | NA NA | Low |
| Huitsing et al. (2020) | KiVa (NL, NL+) | Netherlands | 4724 | Primary school | 8.66 | 0.69 |  | Girls | 2405 | Low  Middle | NA NA | Low |
| Palladino et al. (2016) | NoTrap! | Italy | 622 | High school | 14.58 | 0.88 |  | Girls | 245 | Low  Middle | NA NA | Moderate |
| Cross et al. (2011) | Friendly Schools | Australia | 1968 | Primary school | 8.56 | 0.55 |  | Girls | 976 | Low  Middle | 19.8 40.8 | Moderate |
| Kärna et al. (2011) & Juvonen et al. (2016) & Salmivalli et al. (2005) | KiVa | Finland | 8237 | Primary school | 11.00 | 1.11 |  | Girls | 9527 | Low  Middle | NA NA | Moderate |
| Kärna et al. (2013) | KiVa | Finland | 19191 | Middle school | 14.36 | 0.89 |  | Girls | 4115 | Low Middle | NA NA | Moderate |

*Note.* SES = Social-economic status; NA = Not available.

**Table 2**

*Included Components of Anti-Bullying Interventions.*

|  | School policy | Monitor | Class rules | School assemblies | Student placement | Peer involvement | Playground supervision | Solely non-punitive disciplinary methods | Both non-punitive and punitive disciplinary methods | Psychoeducation | Cognitive emotional skill-building |
| --- | --- | --- | --- | --- | --- | --- | --- | --- | --- | --- | --- |
| ViSC | * |  | * |  |  |  |  | * |  | * | * |
| Friendly Attack |  |  |  |  |  |  |  |  |  | * |  |
| Drama program |  |  |  |  |  |  |  |  |  | * | * |
| WITS programs | * |  | * | * |  |  | * | * |  | * | * |
| KiVa (IT) | * |  | * | * |  |  | * |  | * | * | * |
| KiVa (NL) | * |  | * | * |  |  | * | * |  | * | * |
| KiVa+ (NL) | * | * | * | * |  |  | * | * |  | * | * |
| NoTrap! |  |  |  |  |  | * |  |  |  | * | * |
| Friendly schools | * | * | * | * | * |  | * |  | * | * | * |
| KiVa (original) | * |  | * | * |  |  | * |  | * | * | * |

**S5.** Harmonization of Bullying and Victimization Measures

To harmonize different outcome measures into single 5-point scores for victimization and bullying perpetration, we (1) used all studies that employed the one-item, 5-point Likert scale to obtain the percentile distribution across the score categories, (2) calculated the percentile thresholds for the sum scores per study that used a multiple-item outcome, and (3) used these percentile thresholds to transform the sum score into a 5-point score. To examine if our harmonization approach was successful, we calculated correlations and chi-square coefficients to assess the association and agreement between the original and transformed outcome measure in four studies that used both the one-item and multiple-item bullying and victimization measures. The transformed 5-point outcome was moderately correlated (*r*s = .53 to .66) with the original (1 item) 5-point outcome measure and had good weighted agreement—defined as the percentage of scores falling in the same score category or one category off—between the transformed and original scale of 0.88 to 0.95.

**Table 1.**

*Cumulative percentage distribution of the pre- and post-intervention 5 point scale.*

| **Measure** | 0 | 1 | 2 | 3 | 4 |
| --- | --- | --- | --- | --- | --- |
| Victim pre | 60 | 84 | 90 | 94 | 100 |
| Victim post | 64 | 87 | 92 | 96 | 100 |
| Bully pre | 68 | 92 | 96 | 98 | 100 |
| Bully post | 73 | 93 | 97 | 98 | 100 |

**Table 2.**

*Threshold values for the sum scores to transform to the 5 point scale for each trial.*

| **Measure** | Trial 5 | Trial 7 | Trial 11 | Trial 1 | Trial 6 | Trial 4 |
| --- | --- | --- | --- | --- | --- | --- |
| Victim pre | 3-8-10-15-52 | 3-6-8-10-27 | 1-4-6-8-42 | 3-8-11-15-44 | 5-13-17-22-40 | 4-8-10-13-20 |
| Victim post | 3-8-11-15-38 | 2-5-6-8-23 | 1-4-6-10-42 | 4-12-16-22-44 | 3-10-13-18-40 | 4-8-9-11-20 |
| Bully pre | 2-6-8-13-56 | 4-10-16-21-37 | 1-5-8-12-42 | 3-10-15-22-44 | 1-8-15-21-40 | NA |
| Bully post | 2-6-9-12-32 | 4-10-14-15-23 | 1-5-10-14-42 | 4-15-22-25-44 | 0-4-11-16-40 | NA |

**Table 3.**

*Comparing the observed 5pt scores to the transformed scores.*

| **Measure** | Correlation 5pt to sum score | Correlation 5pt to transformed | Agreement^a^ | Weighted agreement^b^ |
| --- | --- | --- | --- | --- |
| Victim pre | 0.654 | 0.577 | 0.62 | 0.88 |
| Victim post | 0.661 | 0.621 | 0.69 | 0.92 |
| Bully pre | 0.536 | 0.558 | 0.70 | 0.94 |
| Bully post | 0.575 | 0.584 | 0.76 | 0.95 |

*Note: ^a^Agreement is the percentage of the scores that fall in the same category in both the measured 5pt score and the transformed 5pt score. ^b^Weigthed agreement is the percentage of the scores that fall in the same category or 1 category off.*

**Table 4.**

*Cumulative percentage distribution of the transformed pre- and post-intervention 5 point scale*

| **Measure** | 0 | 1 | 2 | 3 | 4 |
| --- | --- | --- | --- | --- | --- |
| Victim pre scale | 64 | 86 | 91 | 95 | 100 |
| Victim post scale | 70 | 88 | 92 | 96 | 100 |
| Bully pre scale | 73 | 93 | 96 | 98 | 100 |
| Bully post scale | 77 | 94 | 97 | 98 | 100 |

**S6. ROBINS-I Risk of Bias and Risk of Publication Bias**

**Table 1**.

*Overview of risk of bias of the included trials based on the ROBINS-I.*

| **Trial** | **Overall Risk of Bias** | Participant selection | Classification of interventions | Deviations from intended interventions | Missing data | Measurement of outcomes |
| --- | --- | --- | --- | --- | --- | --- |
| Solomontos-Kountouri et al. (2016) | Moderate | Moderate | Low | Low | Moderate | Low |
| DeSmet et al. (2018) | Moderate | Low | Low | Low | Moderate | Moderate |
| Joronen et al. (2011) | Moderate | Low | Low | Moderate | Moderate | Low |
| Leadbeater et al. (2012) | Moderate | Moderate | Low | Moderate | Moderate | Low |
| Nocentini & Menesini (2016) | Low | Low | Low | Low | Low | Low |
| Huitsing et al. (2020) | Low | Low | Low | Low | Low | Low |
| Palladino et al. (2016) | Moderate | Moderate | Low | Moderate | Moderate | Low |
| Cross et al. (2011) | Moderate | Low | Low | Low | Moderate | Low |
| Kärna et al. (2011), Juvonen et al. (2016), Salmivalli et al. (2005) | Moderate | Low | Low | Moderate | Low | Low |
| Kärna et al. (2013) | Moderate | Low | Low | Low | Moderate | Low |

**Table 2**.

*Overview of studies selected after screening for which researchers were contacted to request full datasets.*

| **Data shared** | | | | | **Data not shared** | | | | |
| --- | --- | --- | --- | --- | --- | --- | --- | --- | --- |
| Study# | PubYear | Location | Design | Reported effects | Study# | PubYear | Location | Design | Reported effects |
| 1 | 2016 | Cyprus | Quasi-experimental | **CODE = 1.1**   - Significant quadratic effects victimization and perpetration (steeper increase c/w control but over time this increase became smaller in intervention compared with control) - Small to medium effects. | 14 | 2017 | Turkey | RCT | **CODE = 4**   - Non-significant change in intervention group on bullying behavior. - Significant change in intervention group on victimization. |
| 2 | 2018 | Belgium | Cluster RCT | **CODE = 2**   - No significant effects on (cyber-)bullying victimization or perpetration   ES_range_: -0.15; 0.09). | 15 | 2007 | United States | Quasi-experimental | **CODE = 2**   - Non-significant changes in intervention group on victimization. |
| 3 | 2011 | Finland | Quasi-experimental | **CODE = 2**   - Reduction (1.6 – 5.9%) in bulling behavior, non-significant difference between intervention and control group. - Reduction (1.6 – 20.7%) in victimization, non-significant difference between intervention and control group. | 16 | 2016 | Sweden | Quasi-experimental | **CODE = 2**   - Non-significant changes in intervention group on victimization. |
| 4^c^ | 2016 | Canada | RCT | - No direct effects of the intervention on victimization reported. | 17 | 2018 | UK | RCT | **CODE = 1.3**   - Significant changes in intervention group on victimization (ES_range_: -.05 to -.08) |
| 5 | 2016 | Italy | RCT | **CODE = 1.1**   - Victimization and bullying decreased significantly over time in intervention groups - (ES_range_: .21 to .38). | 18 | 2016 | Germany | RCT | **CODE = 4**   - Significant changes in intervention group on traditional bullying and cyberbullying behavior (ES_range_: -.25 to -.27). - Non-significant effect of intervention on victimization. |
| 6 | 2020 | Netherlands | RCT | **CODE = 1.1**   - Victimization and bullying reduced more strongly in intervention schools compared with control schools, with stronger effects after two school years than after one school year of implementation - The odds for intervention students to be victimized or to bully were 1.34 and 1.67 lower than for control students (after two intervention years) | 19 | 2017 | Turkey | Quasi-experimental | **CODE = 1.1**   - Significant change in intervention groups on bullying behavior and victimization. |
| 7 | 2016 | Italy | Quasi-experimental | **CODE = 1.1**  Trial 1   - Significant decrease of victimization and bullying over time in intervention group     Trial 2   - Significant decrease of victimization and bullying over time in intervention group - (ES_range_: .25 to .26). | 20 | 2017 | New Zealand | RCT | **CODE = 2**   - Non-significant change in intervention group on bullying behavior (child report). |
| 8^a^ | 2015 | United States | Quasi- experimental | **CODE = 2**   - No significant treatment effects were identified for bullying perpetration and victimization | 21 | 2018 | Spain | RCT | **CODE = 1.3**   - Significant change in bullying victimization in the intervention group (ES = .60). |
| 9 | 2011 | Australia | RCT | **CODE = 1.3**   - Significant change in intervention group on victimization | 22/37 | 2014/ 2016 | Austria | RCT | **CODE = 1.1**   - program is effective in preventing cyberbullying and cyber-victimization and the effects are sustainable after 6 months. |
| 10/11/13 | 2005/  2011/  2016 | Finland | RCT | **CODE = 1.1**   - Only significant intervention effects on bullying and victimization in grade 4 (in expected direction) - (ES_range_: -56% to -79%) in high level implementation schools - Strongest intervention effects for children with higher baseline levels of victimization. - Significant intervention effects on self-reported bullying behavior and victimization at 9-month follow-up (ES_range_: .10 to 17). | 23/41 | 2003/ 2012 | Canada | Quasi-experimental | **CODE = 1.3**   - Significant decrease in physical and relational victimization in the intervention group (ES_range_: .17 to 20). - Intervention moderately related to decreases in classroom levels of victimization |
| 12 | 2013 | Finland | RCT | **CODE = 4**   - Significant intervention effects on bullying behavior and victimization in Grades 1-3.   Non-significant intervention effects on bullying behavior and victimization in Grades 8-9. | 24/25 | 2007/ 2010 | United States | RCT | **CODE = 4**   - Non-significant change in bullying in intervention group (OR = 1.16). - Non-significant change in victimization in intervention group (OR = 1.12). - Significant reduction in victimization in intervention group compared to control group at 12-month follow-up. - Non-significant reduction in bullying behavior in the intervention group compared to the control group at 12-month follow-up. |
| 39^b^ | 2009 | United States | Cluster randomized design | **CODE = 4**   - Universal intervention was associated with reductions in victimization; the selective intervention was not associated with changes in victimization. | 26 | 2014 | Cyprus/Greece | RCT | **CODE = 1.1**   - Significant effect of intervention of bullying behavior and victimization (ES_range_: .46 to 70) |
| 42 | 2011 | Canada | Quasi-experimental | **CODE = 1.3**   - Children in the program showed more rapid declines in peer victimization over time compared with children in control schools. | 27 | 2018 | United States | Quasi-experimental (extended age cohort) | **CODE = 1.1**   - Significant reductions in victimization and bullying - Large to very large - Less effective/ non-significant in some grades |
|  |  |  |  |  | 28^d^ | 2012 | United States | Quasi-experimental | **CODE = 1.1**   - Perpetrating and being victimized **by physical and relational aggression** were statistically significantly lower, in the treatment than in the comparison schools. - Relatively weak in terms of effect size, explaining only 3% of the variance in the outcomes. |
|  |  |  |  |  | 29 | 2010 | UK and Germany | Non-randomized controlled trial | **CODE = 4**   - 26% decrease in victimization risk in the intervention group compared to the control group but only at follow-up 1 (further analysis showed this was only the case for UK students) - No difference in bullying perpetration among students |
|  |  |  |  |  | 30 | 2018 | Italy | Experimental design | **CODE = 4**   - Significant decrease both in cyberbullying and cybervictimization among students who received the intervention with a follow-up period of six months. |
|  |  |  |  |  | 31 | 2013 | Netherlands | RCT | **CODE = 1.1**   - Assessed risk-groups effects - The results indicated that the intervention is effective for some children but less for others |
|  |  |  |  |  | 32 | 2015 | Romania | Quasi-experimental | **CODE = 2**   - No behavioral change was found in the 2 experimental groups when compared with the control group. |
|  |  |  |  |  | 33 | 2013 | Finland | RCT | **CODE = 4**   - Significant intervention effect on cybervictimization; odds of students in the control condition reporting more frequent cybervictimization were 29% greater than the odds of students in the intervention conditions. Cohen's *d* = .14 - Effect of the intervention on cyberbullying varied as a function of student's age – only sig. effective for younger students. Cohen's *d* = .03 |
|  |  |  |  |  | 34 & 40 | 2014/ 2015 | Germany | Pre-post test (randomly assigned classes within schools) | **CODE = 1.2**   - Reduced cyberbullying behavior within intervention classes compared with control group - only for long-term intervention; ES = -.64 |
|  |  |  |  |  | 35 | 2011 | China | Quasi-experimental | **CODE = 1.2**   - Full intervention group had significant reduction of bullying, compared with partial intervention and the control group (ES = .18) |
|  |  |  |  |  | 36 | 2012 | China | Quasi-experimental | **CODE = 1.1**   - Highly significant main effect: reduction in victimization/ bullying in intervention schools (F = 7.70). Most significant reductions occurred when a whole-school intervention was used (F = 10.73). - Composite score of both victimization and perpetration |
|  |  |  |  |  | 38 | 2007 | Australia | Randomized prospective design | **CODE = 4**   - No differences in the degree to which reported victimization changed over time between students in the ‘intervention’ versus ‘control’ schools. - Significant difference between the ‘control’ and ‘intervention’ schools in the number of students who reported having bullied others (only for boys in 1 school). |

*Note.* Only the results on self-reported bullying behavior and victimization reported in the included and not included papers are reported in this table. Studies may have included additional variables. CODE = 1.1: Significant intervention effects in expected direction reported for both bullying behavior and victimization; CODE = 1.2: Significant intervention effects in expected direction reported for bullying behavior only; CODE = 1.3: Significant intervention effects in expected direction reported for victimization only; CODE = 2: Non-significant effects reported; CODE = 3: Significant intervention effects in unexpected direction (i.e., increased bullying behavior/victimization) reported; CODE = 4: Mixed intervention effects reported.

^a^ This study was not included in the final dataset because researchers provided a different dataset that was not identified through screening and at the time of checking and identifying the difference it was too far in the process to add new datasets.

^b^ This study was not included in the final dataset because upon receiving the data the intervention appeared not to fit our scope.

^c^  This study was not included in the final dataset due to a miscommunication.

^d^ The full dataset from this study was requested, but the outcome measure did not fit our scope. Thus, this study was excluded after a second full-text screening.

**Publication bias analysis**

*Year of publication*

- Studies for which data was requested and shared were published between 2005 and 2020.
- Studies that data was requested for but not shared were published between 2003 and 2018.
- Exclusion of studies 4, 8, 39, and 28 did not change the range of publication dates.

*Location of study (by continent)*

- Of the studies for which data was requested and shared 61.54% of the data were gathered in Europe, 0% in Asia, 0% in Africa, 30.77% in America, and 7.69% in Oceania.
- Of the studies for which data was requested but not shared 64% of the data were gathered in Europe, 8% in Asia, 0% in Africa, 20% in America, and 8% in Oceania
- After exclusion of studies 4, 8, 39, and 28, which were all conducted in America, the percentages changed: Of the studies for which data was requested and shared, 80% was gathered in Europe (vs. 66.67% for studies that did not share data), 0% in Asia (vs. 8.33% for studies that did not share data), 0% in Africa (which is similar for studies that did not share data), 10% in America (vs. 16.67% for studies that did not share data), and 10% in Oceania (vs. 8.33% for studies that did not share data).

*Design*

- five papers (38.46%) for which data was requested and shared had a quasi-experimental design, and eight had a RCT design (61.54%).
- 13 papers (56.52%) for which data was requested but not shared had a quasi-experimental design, and ten had a RCT design (43.48%).
- After exclusion of studies 4, 8, 39, and 28, for studies for which data was requested and shared, the percentage of studies with a quasi-experimental design was 40% versus 60% with a RCT design, and for studies for which data was requested but not shared, the percentage of studies with a quasi-experimental design was 54.55% versus 45.45% with a RCT design

*Reported effects*

- Seven studies (58.34%) for which data was requested and shared reported significant intervention effects on bullying behavior and/or victimization that were all in the expected direction, three studies (25%) reported non-significant intervention effects, and two studies (16.67%), reported mixed intervention effects. One study did not report direct effects and was thus not given a code.
- 12 studies (52.17%) for which data was requested but not shared reported significant intervention effects on bullying behavior and/or victimization that were in the expected direction, four studies (17.39%) reported non-significant intervention effects, and seven studies (30.43%) reported mixed intervention effects.
- After exclusion of studies 4, 8, 39, and 28, for studies for which data was requested and selected, seven studies (70%) reported significant intervention effects on bullying behavior and/ or victimization that were as expected (vs. 50% for studies for which data was not shared), two studies (20%) reported non-significant intervention effects (vs. 18.18% for studies for which data was not shared), and one study (10%) reported mixed intervention effects (vs. 31.82% for studies for which data was not shared).

*Final judgement of bias*

No significant differences were found between studies that were eligible and shared their data and studies that were eligible and did not share their data on: the continent that they gathered data in, the effects that they reported (i.e., significant and as expected, non-significant, or mixed), and the design that they used (i.e., RCT or quasi-experimental). See Table 3 for test statistics.

It is important to emphasize that the studies included in our IPD did not gather data in Asia or Africa, which might hold implications for the generalizability of our findings to these continents.

**Table 3**

*Chi-Square test assessing differences between studies that were identified as eligible and shared their data and studies that were eligible and did not share their data*

|  | *X^2^* | *df* | *p* |
| --- | --- | --- | --- |
| Full sample (*N*=36) |  |  |  |
| Continent | 1.412 | 3 | .703 |
| Effects | 2.664 | 3 | .446 |
| Design | 1.084 | 1 | .298 |
| After Exclusion (*N*=32) |  |  |  |
| Continent | 5.482 | 6 | .476 |
| Effects | 3.629 | 6 | .727 |
| Design | 1.440 | 2 | .487 |

**S7.** Baseline Frequencies and Proportions of Bullying and Victimization.

**Table 1**

*Baseline Logistic Regression Comparisons between Subgroups on Pretest Victimization and Bullying Perpetration*

| **Victimization Model** | Coefficient | *SE* | *t* | Sig. | Exp (Coefficient) | 95% CI (coef.) |
| --- | --- | --- | --- | --- | --- | --- |
|  |  |  |  |  |  | [LL, UL] |
| Sex | -0.245 | .033 | -7.452 | <.001 | 0.783 | [0.734; 0.835] |
| Age | -0.168 | .020 | -8.239 | <.001 | 0.845 | [0.812; 0.880] |
| Ethnicity | 0.236 | .062 | 3.793 | <.001 | 1.266 | [1.121; 1.429] |
| SES high | -0.541 | .135 | -4.020 | <.001 | 0.582 | [0.447; 0.758] |
| SES medium | -0.223 | .115 | -1.948 | .052 | 0.800 | [0.639; 1.001] |
| Intervention vs. Control | -0.021 | .034 | -0.626 | -.532 | 0.979 | [0.917; 1.046] |
| **Perpetration Model** | Coefficient | *SE* | *t* | Sig. | Exp (Coefficient) | 95% CI (coef.) |
|  |  |  |  |  |  | [LL, UL] |
| Sex | -0.797 | .048 | -16.600 | <.001 | 0.451 | [0.410; 0.495] |
| Age | 0.075 | .030 | 2.511 | .012 | 1.078 | [1.017; 1.144] |
| Ethnicity | 0.283 | .084 | 3.388 | .001 | 1.328 | [1.127; 1.564] |
| SES high | -0.371 | .185 | -2.007 | .045 | 0.690 | [0.480; 0.992] |
| SES medium | -0.092 | .188 | -0.489 | .625 | 0.912 | [0.631; 1.319] |
| Intervention vs. Control | -0.065 | .047 | -1.399 | .162 | 0.937 | [0.855; 1.026] |

**Table 2**

*Post-intervention Logistic Regression Comparisons between Subgroups on Posttest Victimization and Bullying Perpetration*

| **Victimization Model** | Coefficient | *SE* | *t* | Sig. | Exp (Coefficient) | 95% CI (coef.) |
| --- | --- | --- | --- | --- | --- | --- |
|  |  |  |  |  |  | [LL, UL] |
| Sex | -0.258 | .036 | -7.247 | <.001 | 0.772 | [0.720; 0.828] |
| Age | -0.119 | .022 | -5.343 | <.001 | 0.888 | [0.850; 0.927] |
| Ethnicity | 0.201 | .068 | 2.933 | .003 | 1.222 | [1.069; 1.397] |
| SES high | -0.580 | .141 | -4.120 | <.001 | 0.560 | [0.425; 0.738] |
| SES medium | -0.295 | .124 | -2.375 | .018 | 0.745 | [0.584; 0.950] |
| Intervention vs. Control | -0.226 | .036 | -6.235 | <.001 | 0.798 | [0.743; 0.857] |
| Initial victimization | 1.816 | .041 | 43.864 | <.001 | 6.149 | [5.669; 6.669] |
| **Perpetration Model** | Coefficient | *SE* | *t* | Sig. | Exp (Coefficient) | 95% CI (coef.) |
|  |  |  |  |  |  | [LL, UL] |
| Sex | -0.815 | .053 | -15.507 | <.001 | 0.443 | [0.399; 0.491] |
| Age | 0.085 | .033 | 2.604 | .009 | 1.089 | [1.021; 1.161] |
| Ethnicity | 0.322 | .094 | 3.427 | .001 | 1.380 | [1.148; 1.659] |
| SES high | -0.057 | .187 | -0.303 | .762 | 0.945 | [0.655; 1.363] |
| SES medium | -0.065 | .192 | -0.337 | .736 | 0.937 | [0.644; 1.365] |
| Intervention vs. Control | -0.121 | .050 | -2.403 | .016 | 0.886 | [0.803; 0.978] |
| Initial perpetration | 2.138 | .061 | 35.080 | <.001 | 8.480 | [7.525; 9.556] |

**S8.** Forest Plots of Main Analyses (for whom does it work and what works)

**Figure 1**

*Forest Plots of Interaction Effects of Subgroup × Intervention Status on Post-Intervention Victimization (left) and Perpetration (right)*


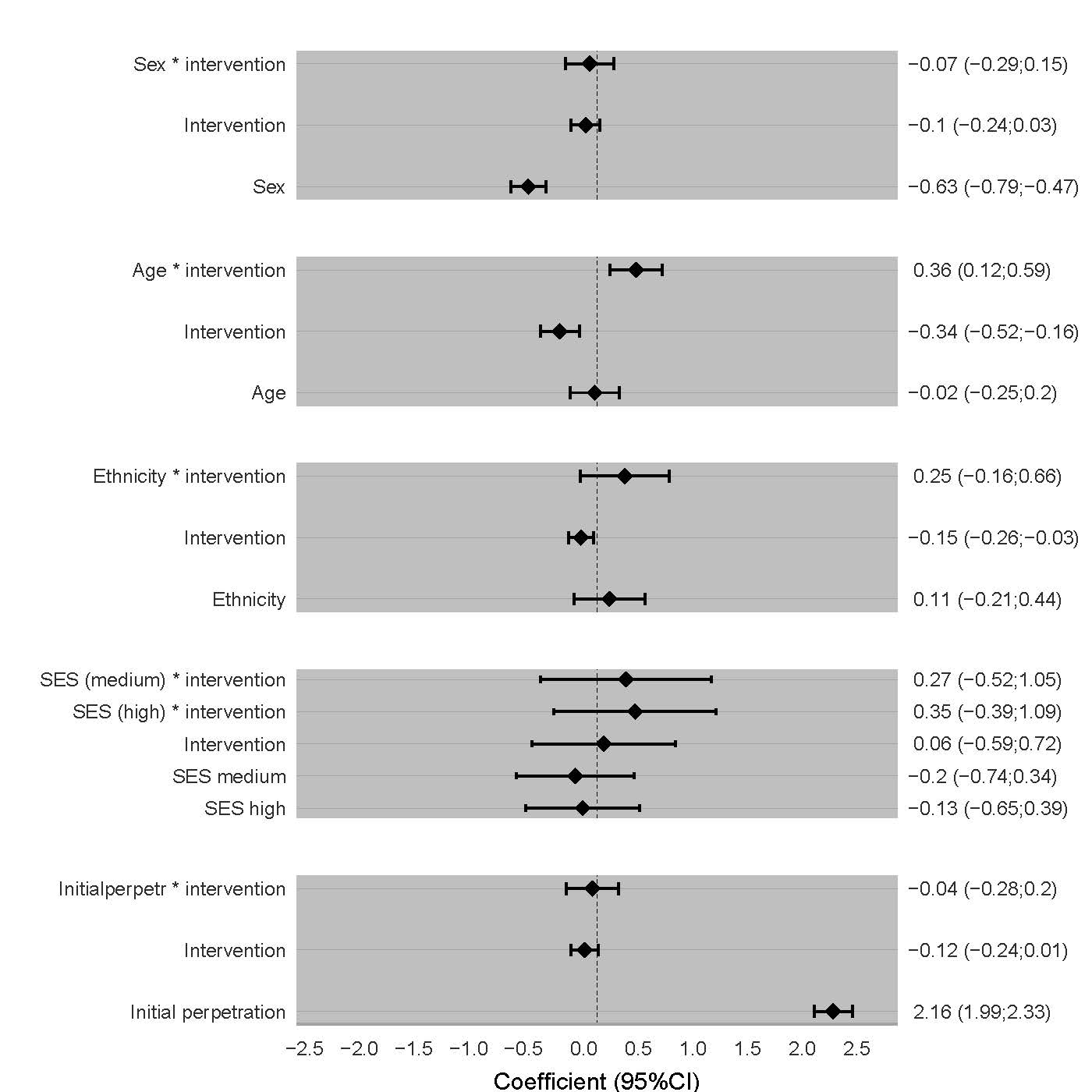


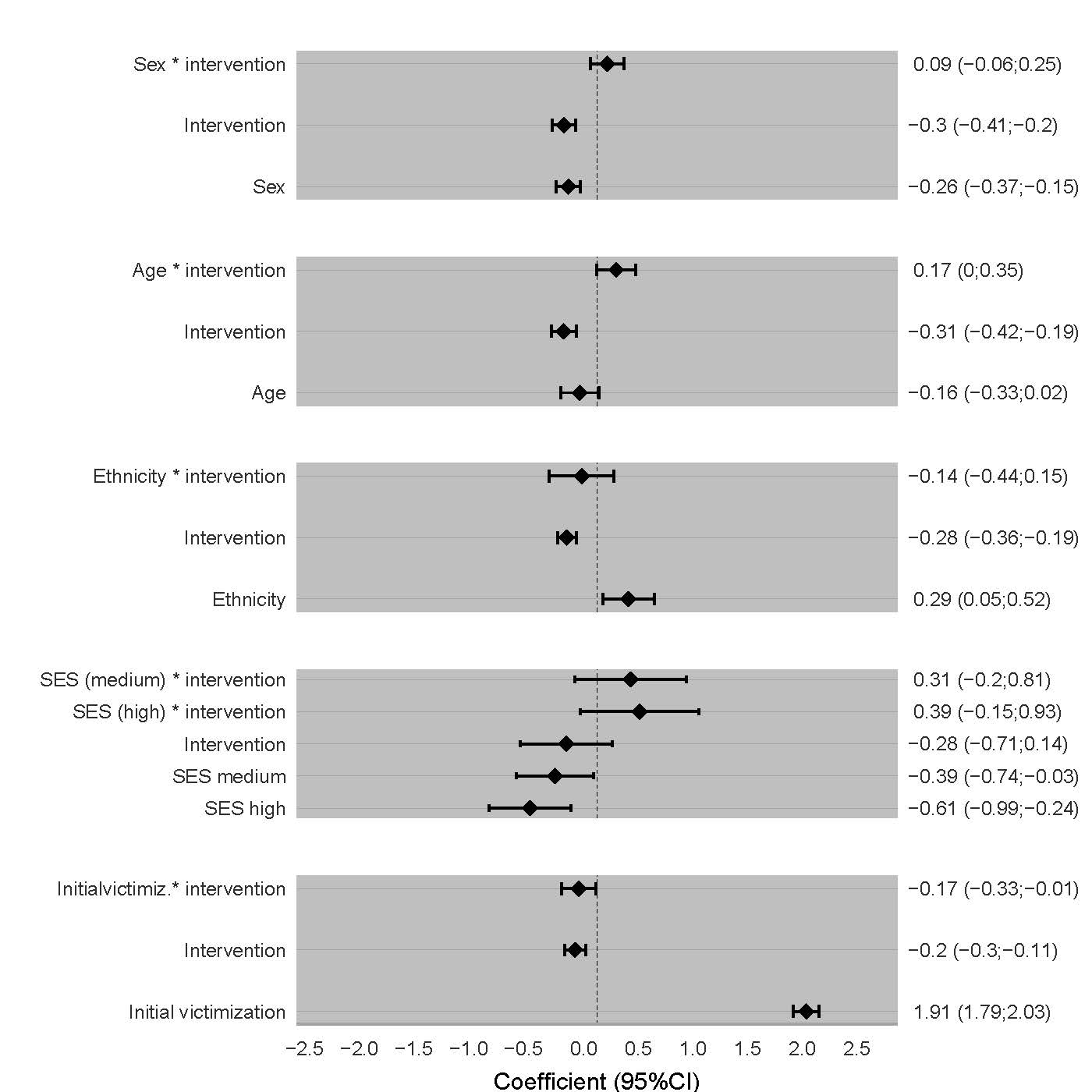


**Figure 2**

*Forest Plots of Main Effects of Intervention Components on Post-Intervention Victimization (left) and Perpetration (right)*


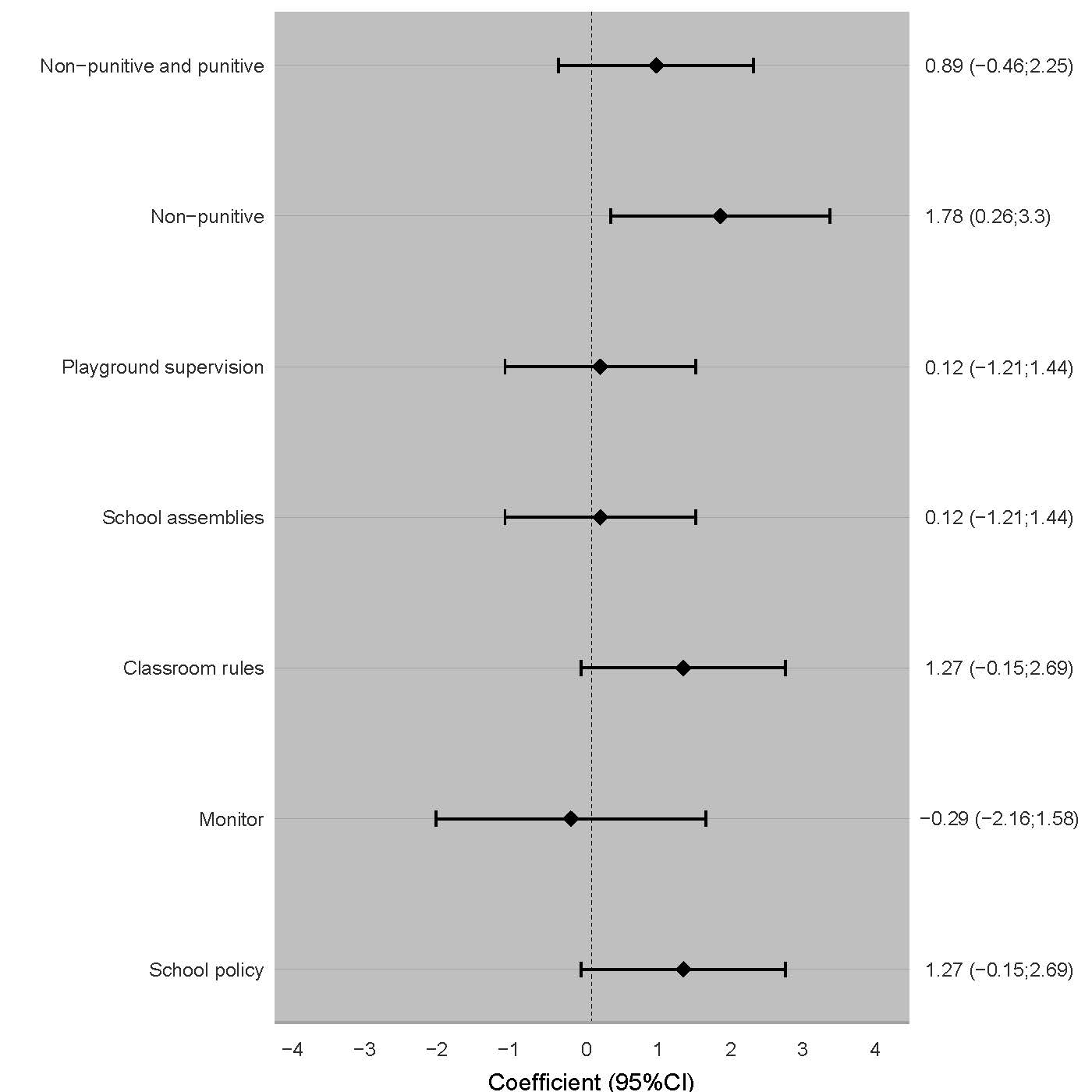


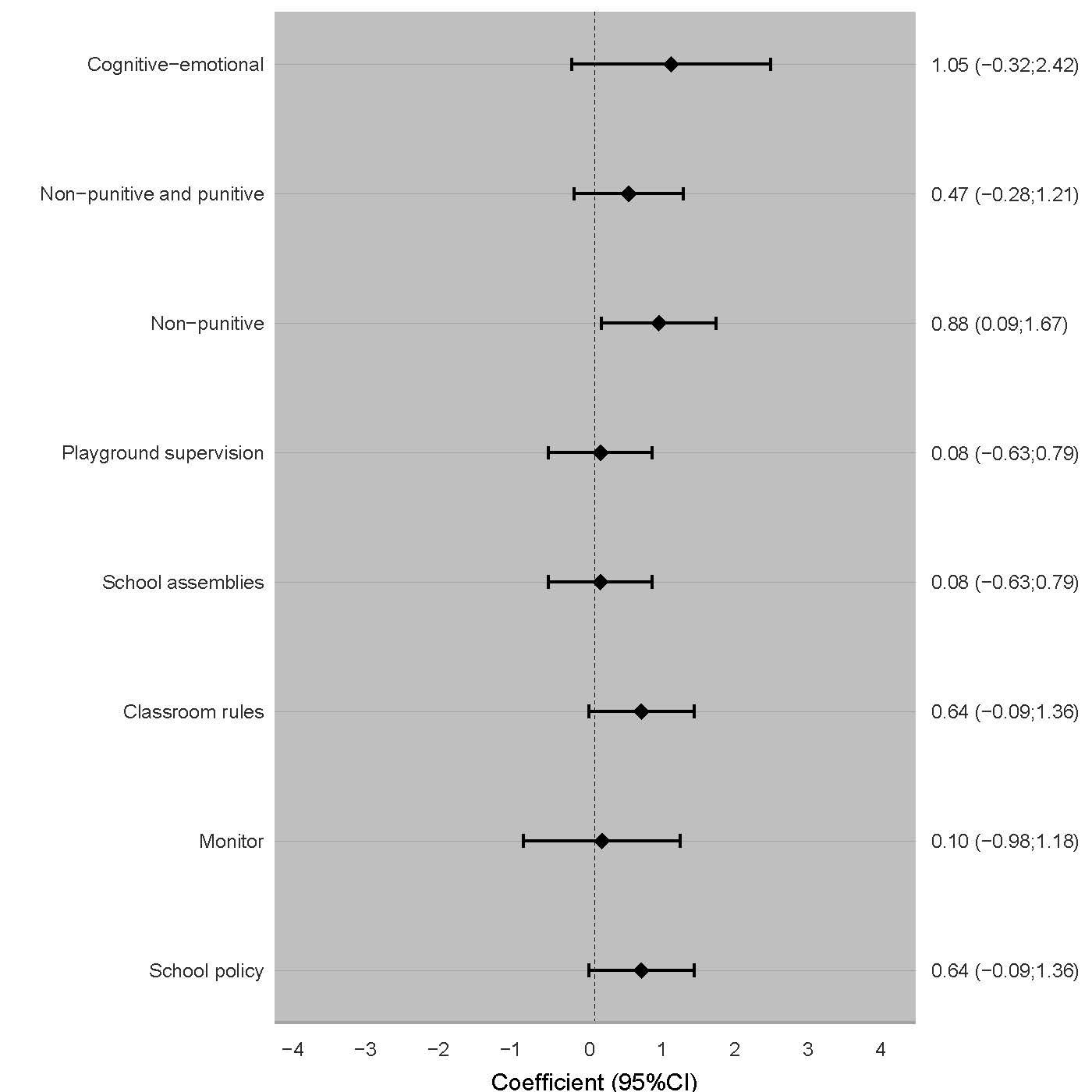


**S9.** Exploratory Analyses: What Works for Whom

**Table 1**

*Interaction Effects of Sex x Intervention Components for Post-Intervention Bullying Victimization and Perpetration*

| **Victimization Model** | Coefficient | | *SE* | *t* | Sig. | Exp (Coefficient) | 95% CI (coef.) | Rank | Adj. *a* |
| --- | --- | --- | --- | --- | --- | --- | --- | --- | --- |
|  |  |  |  |  |  |  | [LL, UL] |  |  |
| Sex | -1.008 | | 0.436 | -2.313 | .021 | 0.365 | [0.16, 0.86] |  |  |
| School policy | 0.302 | | 0.395 | 0.765 | .444 | 1.352 | [0.62, 2.93] |  |  |
| School policy * sex | 0.848 | | 0.439 | 1.931 | .054 | 2.334 | [0.99, 5.52] | 3 | .094 |
| Sex | -0.186 | | 0.055 | -3.397 | .001 | 0.831 | [0.75, 0.92] |  |  |
| Monitor | 0.008 | | 0.561 | 0.015 | .988 | 1.008 | [0.34, 3.03] |  |  |
| Monitor * sex | 0.179 | | 0.218 | 0.823 | .411 | 1.196 | [0.78, 1.83] | 7 | .219 |
| Sex | -1.008 | | 0.436 | -2.313 | .021 | 0.365 | [0.16, 0.86] |  |  |
| Classroom rules | 0.302 | | 0.395 | 0.765 | .444 | 1.352 | [0.62, 2.93] |  |  |
| Classroom rules * sex | 0.848 | | 0.439 | 1.931 | .054 | 2.334 | [0.99, 5.52] | 2 | .063 |
| Sex | -0.437 | | 0.195 | -2.236 | .025 | 0.646 | [0.41, 0.95] |  |  |
| School assemblies | -0.059 | | 0.374 | -0.157 | .875 | 0.943 | [0.45, 1.96] |  |  |
| School assemblies * sex | 0.283 | | 0.203 | 1.396 | .163 | 1.328 | [0.89, 1.97] | 6 | .186 |
| Sex | -0.437 | | 0.195 | -2.236 | .025 | 0.646 | [0.44, 0.95] |  |  |
| Playground supervision | -0.059 | | 0.374 | -0.157 | .875 | 0.943 | [0.45, 1.96] |  |  |
| Playground supervision * sex | 0.283 | | 0.203 | 1.396 | .163 | 1.328 | [0.89, 1.98] | 5 | .156 |
| Sex | 1.008 | | 0.436 | -2.315 | .021 | 0.365 | [0.16, 0.86] |  |  |
| Non punitive | 0.456 | | 0.426 | 1.068 | .285 | 1.577 | [0.68, 3.64] |  |  |
| Non punitive and punitive | 0.179 | | 0.406 | 0.440 | .660 | 1.196 | [0.54, 2.65] |  |  |
| Non-punitive * sex | 1.029 | | 0.444 | 2.316 | .021 | 2.799 | [1.17, 6.69] | 1 | .031 |
| Non-punitive and punitive * sex | 0.740 | | 0.441 | 1.679 | .093 | 2.097 | [0.88, 4.98] | 4 | .125 |
| Sex | -0.311 | | 1.025 | -0.303 | .762 | 0.733 | [0.10, 5.47] |  |  |
| Cognitive-emotional | 0.951 | | 0.870 | 1.093 | .275 | 2.587 | [0.47, 14.24] |  |  |
| Cognitive-emotional * sex | 0.137 | | 1.027 | 0.134 | .894 | 1.147 | [0.15, 8.58] | 8 | .250 |
| **Perpetration Model** | Coefficient | | *SE* | *t* | Sig. | Exp (Coefficient) | 95% CI (coef.) | Rank | Adj. *a* |
|  |  |  |  |  |  |  | [LL, UL] |  |  |
| Sex | 0.005 | 0.546 | | 0.009 | .992 | 1.005 | [0.35, 2.93] |  |  |
| School policy | 1.593 | 0.779 | | 2.045 | .041 | 4.918 | [1.07, 22.65] |  |  |
| School policy * sex | -0.737 | 0.551 | | -1.337 | .181 | 0.479 | [0.16, 1.41] | 4 | .143 |
| Sex | -0.690 | 0.080 | | -8.589 | <.001 | 0.502 | [0.43, 0.59] |  |  |
| Monitor | -0.066 | 0.976 | | -0.067 | .946 | 0.936 | [0.14, 6.35] |  |  |
| Monitor * sex | -0.877 | 0.506 | | -1.736 | .083 | 0.416 | [0.15, 1.12] | 1 | .036 |
| Sex | 0.005 | 0.546 | | 0.009 | .992 | 1.005 | [0.35, 2.93] |  |  |
| Classroom rules | 1.593 | 0.779 | | 2.045 | .041 | 4.918 | [1.07, 22.65] |  |  |
| Classroom rules * sex | -0.737 | 0.551 | | -1.337 | .181 | 0.479 | [0.16, 1.41] | 3 | .107 |
| Sex | -0.585 | 0.211 | | -2.770 | .006 | 0.557 | [0.37, 0.84] |  |  |
| School assemblies | 0.174 | 0.689 | | 0.253 | .800 | 1.191 | [0.31, 4.60] |  |  |
| School assemblies * sex | -0.154 | 0.228 | | -0.675 | .499 | 0.857 | [0.55, 1.34] | 7 | .250 |
| Sex | -0.585 | 0.211 | | -2.770 | .006 | 0.557 | [0.37, 0.84] |  |  |
| Playground supervision | 0.174 | 0.689 | | 0.253 | .800 | 1.191 | [0.31, 4.59] |  |  |
| Playground supervision * sex | -0.154 | 0.228 | | -0.675 | .499 | 0.857 | [0.55, 1.34] | 6 | .214 |
| Sex | -0.004 | 0.545 | | -0.007 | .995 | 0.996 | [0.34, 2.90] |  |  |
| Non-punitive | 2.040 | 0.826 | | 2.469 | .014 | 7.691 | [1.52, 38.86] |  |  |
| Non-punitive and punitive | 1.228 | 0.742 | | 1.655 | .098 | 3.415 | [0.80, 14.63] |  |  |
| Non-punitive * sex | -0.541 | 0.562 | | -0.964 | .335 | 0.582 | [0.19, 1.75] | 5 | .179 |
| Non-punitive and punitive * sex | -0.825 | 0.554 | | -1.488 | .137 | 0.438 | [0.15, 1.30] | 2 | .071 |

**Table 2**

*Interaction Effects of Age x Intervention Components for Post-Intervention Bullying Victimization and Perpetration*

| **Victimization Model** | Coefficient | *SE* | *t* | Sig. | Exp (Coefficient) | 95% CI (coef.) | Rank | Adj. *a* |
| --- | --- | --- | --- | --- | --- | --- | --- | --- |
|  |  |  |  |  |  | [LL, UL] |  |  |
| Age | -0.847 | 0.755 | -1.122 | .262 | 0.429 | [0.10, 1.88] |  |  |
| School policy | -0.055 | 0.659 | -0.083 | .934 | 0.947 | [0.26, 3.44] |  |  |
| School policy * age | 0.953 | 0.763 | 1.250 | .211 | 2.594 | [0.58, 11.57] | 2 | .083 |
| Age | -0.847 | 0.755 | -1.122 | .262 | 0.429 | [0.10, 1.88] |  |  |
| Classroom rules | -0.055 | 0.659 | -0.083 | .934 | 0.947 | [0.26, 3.44] |  |  |
| Classroom rules * age | 0.953 | 0.763 | 1.250 | .211 | 2.594 | [0.58, 11.57] | 3 | .125 |
| Age | -0.040 | 0.126 | -0.315 | .753 | 0.961 | [0.75, 1.23] |  |  |
| School assemblies | 0.009 | 1.716 | 0.005 | .966 | 1.009 | [0.04, 29.17] |  |  |
| School assemblies * age | -0.012 | 0.131 | -0.093 | .926 | 0.988 | [0.76, 1.28] | 6 | .250 |
| Age | 0.166 | 0.225 | 0.738 | .461 | 1.181 | [0.76, 1.84] |  |  |
| Playground supervision | 0.095 | 0.438 | 0.217 | .828 | 1.100 | [0.47, 2.60] |  |  |
| Playground supervision * age | -0.113 | 0.259 | -0.437 | .662 | 0.893 | [0.54, 1.48] | 5 | .208 |
| Age | -0.842 | 0.715 | -1.177 | .239 | 0.431 | [0.11, 1.75] |  |  |
| Non-punitive | 0.181 | 0.679 | 0.267 | .790 | 1.199 | [0.32, 4.54] |  |  |
| Non-punitive and punitive | -0.176 | 0.637 | -0.276 | .782 | 0.839 | [0.24, 2.92] |  |  |
| Non-punitive *age | 1.163 | 0.751 | 1.548 | .122 | 3.201 | [0.73, 13.96] | 1 | .042 |
| Non-punitive and punitive*age | 0.880 | 0.727 | 1.211 | .226 | 2.411 | [0.58, 10.02] | 4 | .167 |
| **Perpetration Model** | Coefficient | *SE* | *t* | Sig. | Exp (Coefficient) | 95% CI (coef.) | Rank | Adj. *a* |
|  |  |  |  |  |  | [LL, UL] |  |  |
| Age | 0.471 | 0.235 | 2.001 | .045 | 1.601 | [1.01, 2.54] |  |  |
| Playground supervision | 0.477 | 0.851 | 0.560 | .575 | 1.611 | [0.30, 8.55] |  |  |
| Playground supervision * age | -0.322 | 0.311 | -1.034 | .301 | 0.725 | [0.39, 1.33] | 1 | .083 |
| Age | 0.434 | 0.804 | 0.539 | .590 | 1.543 | [0.32, 7.47] |  |  |
| Non-punitive | 1.597 | 0.793 | 2.012 | .044 | 4.936 | [1.04, 23.28] |  |  |
| Non-punitive and punitive | 0.516 | 0.775 | 0.666 | .505 | 1.676 | [0.37, 7.66] |  |  |
| Non-punitive *age | 0.025 | 0.837 | 0.030 | .976 | 1.026 | [0.20, 5.29] | 3 | .250 |
| Non-punitive and punitive*age | -0.318 | 0.809 | -0.393 | .694 | 0.727 | [0.15, 3.55] | 2 | .167 |

**Table 3**

*Interaction Effects of Ethnicity x Intervention Components for Post-Intervention Bullying Victimization and Perpetration*

| **Victimization Model** | Coefficient | *SE* | *t* | Sig. | Exp (Coefficient) | 95% CI (coef.) | Rank | Adj. *a* |
| --- | --- | --- | --- | --- | --- | --- | --- | --- |
|  |  |  |  |  |  | [LL, UL] |  |  |
| Ethnicity | 0.147 | 0.423 | 0.348 | .728 | 1.158 | [0.51, 2.66] |  |  |
| School assemblies | -0.102 | 0.606 | -0.168 | .867 | 0.903 | [0.27, 2.97] |  |  |
| School assemblies * ethnicity | 0.034 | 0.435 | 0.078 | .938 | 1.035 | [0.44, 2.43] | 1 | .125 |
| Ethnicity | 0.147 | 0.423 | 0.348 | .728 | 1.158 | [0.51, 2.66] |  |  |
| Playground supervision | -0.102 | 0.606 | -0.168 | .867 | 0.903 | [0.28, 2.96] |  |  |
| Playground superv. * ethnicity | 0.034 | 0.435 | 0.078 | .938 | 1.035 | [0.44, 2.43] | 2 | .250 |
| **Perpetration Model** | Coefficient | *SE* | *t* | Sig. | Exp (Coefficient) | 95% CI (coef.) |  |  |
|  |  |  |  |  |  | [LL, UL] |  |  |
| Ethnicity | 0.676 | 0.399 | 1.694 | .090 | 1.967 | [0.89, 4.30] |  |  |
| School assemblies | -0.042 | 1.016 | -0.041 | .967 | 0.959 | [0.13, 7.03] |  |  |
| School assemblies * ethnicity | -0.360 | 0.424 | -0.851 | .395 | 0.698 | [0.30, 1.60] | 1 | .125 |
| Ethnicity | 0.676 | 0.399 | 1.694 | .090 | 1.967 | [0.90, 4.30] |  |  |
| Playground superv. | -0.042 | 1.016 | -0.041 | .967 | 0.959 | [0.13, 7.03] |  |  |
| Playground superv. * ethnicity | -0.360 | 0.424 | -0.851 | .395 | 0.698 | [0.30, 1.60] | 2 | .250 |

**Table 4**

*Interaction Effects of SES x Intervention Components for Post-Intervention Bullying Victimization and Perpetration*

| **Victimization Model** | Coefficient | *SE* | *t* | Sig. | | Exp (Coefficient) | 95% CI (coef.) | Rank | Adj. *a* |
| --- | --- | --- | --- | --- | --- | --- | --- | --- | --- |
|  |  |  |  |  |  |  | [LL, UL] |  |  |
| SES high | -0.392 | 0.280 | -1.403 | | .161 | 0.675 | [0.40, 1.17] |  |  |
| SES medium | -0.086 | 0.255 | -0.335 | | .737 | 0.918 | [0.56, 1.51] |  |  |
| Monitor | -0.034 | 1.150 | -0.030 | | .976 | 0.966 | [0.10, 9.23] |  |  |
| Monitor * SES High | 0.428 | 0.469 | 0.913 | | .361 | 1.535 | [0.61, 3.85] | 5 | .208 |
| Monitor * SES Medium | -0.007 | 0.379 | -0.018 | | .986 | 0.993 | [0.47, 2.09] | 6 | .250 |
| SES high | -0.687 | 0.310 | -2.215 | | .027 | 0.503 | [0.27, 0.92] |  |  |
| SES medium | -0.527 | 0.337 | -1.563 | | .118 | 0.591 | [0.31, 1.14] |  |  |
| School assemblies | -0.560 | 0.999 | -0.561 | | .575 | 0.571 | [0.08, 4.05] |  |  |
| School assemblies * SES High | 0.862 | 0.478 | 1.802 | | .072 | 2.368 | [0.93, 6.05] | 1 | .042 |
| School assemblies * SES Medium | 0.638 | 0.409 | 1.557 | | .120 | 1.892 | [0.85, 4.22] | 3 | .125 |
| SES high | -0.687 | 0.310 | -2.215 | | .027 | 0.503 | [0.27, 0.92] |  |  |
| SES medium | -0.527 | 0.337 | -1.563 | | .118 | 0.591 | [0.31, 1.14] |  |  |
| Playground supervision | -0.560 | 0.999 | -0.561 | | .575 | 0.571 | [0.08, 4.05] |  |  |
| Playground supervision * SES High | 0.862 | 0.478 | 1.802 | | .072 | 2.368 | [0.93, 6.05] | 2 | .083 |
| Playground supervision * SES Medium | 0.638 | 0.409 | 1.557 | | .120 | 1.892 | [0.85, 4.22] | 4 | .167 |
| **Perpetration Model** | Coefficient | *SE* | *t* | | Sig. | Exp (Coefficient) | 95% CI (coef.) | Rank | Adj. *a* |
|  |  |  |  |  |  |  | [LL, UL] |  |  |
| SES high | -0.012 | 1.356 | -0.009 | | .993 | 0.988 | [0.07, 14.13] |  |  |
| SES medium | -0.034 | 1.396 | -0.024 | | .981 | 0.967 | [0.06, 14.96] |  |  |
| Non-punitive | 0.903 | 1.958 | 0.461 | | .645 | 2.466 | [0.05, 114.91] |  |  |
| Non-punitive and punitive | 0.121 | 1.942 | 0.062 | | .950 | 1.129 | [0.03, 50.95] |  |  |
| Non-punitive * SES High | 0.163 | 1.393 | 0.117 | | .907 | 1.177 | [0.08, 18.10] | 1 | .063 |
| Non-punitive and punitive * SES High | -0.064 | 1.383 | -0.046 | | .963 | 0.938 | [0.06, 14.16] | 4 | .250 |
| Non-punitive * SES Medium | -0.082 | 1.438 | -0.057 | | .955 | 0.922 | [0.06, 15.48] | 3 | .188 |
| Non-punitive and punitive * SES Medium | 0.101 | 1.410 | 0.072 | | .943 | 1.106 | [0.07, 17.58] | 2 | .125 |

**Table 5**

*Interaction Effects of Initial Level of Victimization (ISV) x Intervention Components for Post-Intervention Bullying Victimization and Initial Level of Perpetration (ISP) x Intervention Components for Post-Intervention Perpetration*

| **Victimization Model** | Coefficient | *SE* | *t* | Sig. | Exp (Coefficient) | 95% CI (coef.) | Rank | Adj. *a* |
| --- | --- | --- | --- | --- | --- | --- | --- | --- |
|  |  |  |  |  |  | [LL, UL] |  |  |
| ISV | 1.807 | 0.465 | 3.888 | <.001 | 6.090 | [2.45, 15.14] |  |  |
| School policy | 0.650 | 0.386 | 1.684 | .092 | 1.915 | [0.90, 4.08] |  |  |
| School policy * ISV | -0.052 | 0.468 | -0.111 | .911 | 0.949 | [0.38, 2.38] | 8 | .250 |
| ISV | 1.780 | 0.058 | 30.631 | <.001 | 5.932 | [5.29, 6.65] |  |  |
| Monitor | 0.212 | 0.554 | 0.383 | .702 | 1.236 | [0.42, 3.66] |  |  |
| Monitor * ISV | -0.402 | 0.242 | -1.661 | .097 | 0.669 | [0.42, 1.08] | 1 | .031 |
| ISV | 1.807 | 0.465 | 3.888 | <.001 | 6.090 | [2.45, 15.14] |  |  |
| Classroom rules | 0.650 | 0.386 | 1.684 | .092 | 1.915 | [0.90, 4.08] |  |  |
| Classroom rules * ISV | -0.052 | 0.468 | -0.111 | .911 | 0.949 | [0.38, 2.38] | 7 | .219 |
| ISV | 1.677 | 0.218 | 7.693 | <.001 | 5.349 | [3.49, 8.20] |  |  |
| School assemblies | 0.056 | 0.368 | 0.152 | .879 | 1.057 | [0.51, 2.18] |  |  |
| School assemblies * ISV | 0.085 | 0.226 | 0.377 | .706 | 1.089 | [0.70, 1.67] | 6 | .186 |
| ISV | 1.677 | 0.218 | 7.693 | <.001 | 5.349 | [3.49, 8.20] |  |  |
| Playground supervision | 0.056 | 0.368 | 0.152 | .879 | 1.057 | [0.51, 2.18] |  |  |
| Playground supervision * ISV | 0.085 | 0.226 | 0.377 | .706 | 1.089 | [0.70, 1.69] | 5 | .156 |
| ISV | 1.810 | 0.464 | 3.897 | <.001 | 6.110 | [2.46, 15.18] |  |  |
| Non-punitive | 1.045 | 0.412 | 2.535 | .011 | 2.843 | [1.27, 6.38] |  |  |
| Non-punitive and punitive | 0.400 | 0.393 | 1.018 | .309 | 1.491 | [0.69, 3.22] |  |  |
| Non-punitive * ISV | -0.425 | 0.472 | -0.900 | .368 | 0.654 | [0.26, 1.65] | 2 | .063 |
| Non-punitive and punitive * ISV | 0.193 | 0.470 | 0.411 | .681 | 1.213 | [0.48, 3.05] | 4 | .125 |
| ISV | 2.548 | 1.294 | 1.968 | .049 | 12.778 | [1.01, 161.55] |  |  |
| Cognitive-emotional | 1.182 | 0.752 | 1.570 | .116 | 3.259 | [0.75, 14.24] |  |  |
| Cognitive-emotional * ISV | -0.794 | 1.296 | -0.613 | .540 | 0.452 | [0.04, 5.73] | 3 | .094 |
| **Perpetration Model** | Coefficient | *SE* | *t* | Sig. | Exp (Coefficient) | 95% CI (coef.) | Rank | Adj. *a* |
|  |  |  |  |  |  | [LL, UL] |  |  |
| ISP | 0.358 | 1.069 | 0.335 | .737 | 1.431 | [0.18, 11.62] |  |  |
| School policy | 1.126 | 0.740 | 1.522 | .128 | 3.084 | [0.72, 13.16] |  |  |
| School policy * ISP | 1.757 | 1.072 | 1.639 | .101 | 5.794 | [0.71, 47.36] | 5 | .179 |
| ISP | 2.099 | 0.085 | 24.797 | <.001 | 8.160 | [6.91, 9.63] |  |  |
| Monitor | -0.277 | 0.956 | -0.290 | .772 | 0.758 | [0.12, 4.94] |  |  |
| Monitor * ISP | -0.223 | 0.799 | -0.279 | .780 | 0.800 | [0.17, 3.83] | 7 | .250 |
| ISP | 0.358 | 1.069 | 0.335 | .737 | 1.431 | [0.18, 11.62] |  |  |
| Classroom rules | 1.126 | 0.740 | 1.522 | .128 | 3.084 | [0.72, 13.16] |  |  |
| Classroom rules * ISP | 1.757 | 1.072 | 1.639 | .101 | 5.794 | [0.71, 47.36] | 4 | .143 |
| ISP | 1.105 | 0.230 | 4.800 | <.001 | 3.019 | [1.92, 4.74] |  |  |
| School assemblies | -0.014 | 0.741 | -0.019 | .985 | 0.986 | [0.23, 4.21] |  |  |
| School assemblies * ISP | 1.144 | 0.247 | 4.632 | <.001 | 3.139 | [1.93, 5.09] | 1 | .036 |
| ISP | 1.105 | 0.230 | 4.800 | <.001 | 3.019 | [1.92, 4.74] |  |  |
| Playground supervision | -0.014 | 0.741 | -0.019 | .985 | 0.986 | [0.23, 4.21] |  |  |
| Playground supervision * ISP | 1.144 | 0.247 | 4.632 | <.001 | 3.139 | [1.93, 5.09] | 2 | .071 |
| ISP | 0.373 | 1.068 | 0.349 | .727 | 1.452 | [0.18, 11.79] |  |  |
| Non-punitive | 1.829 | 0.795 | 2.300 | .021 | 6.230 | [1.31, 19.61] |  |  |
| Non-punitive and punitive | 0.663 | 0.705 | 0.940 | .347 | 1.940 | [0.49, 7.73] |  |  |
| Non-punitive * ISP | 1.103 | 1.078 | 1.023 | .306 | 3.012 | [0.37, 24.90] | 6 | .214 |
| Non-punitive and punitive * ISP | 2.115 | 1.073 | 1.971 | .049 | 8.293 | [1.01, 68.01] | 3 | .107 |

**Figure 1**

*Interaction Effects (Odds ratios) of Gender× Non-Punitive disciplinary methods on Post-Intervention Bullying Victimization.*


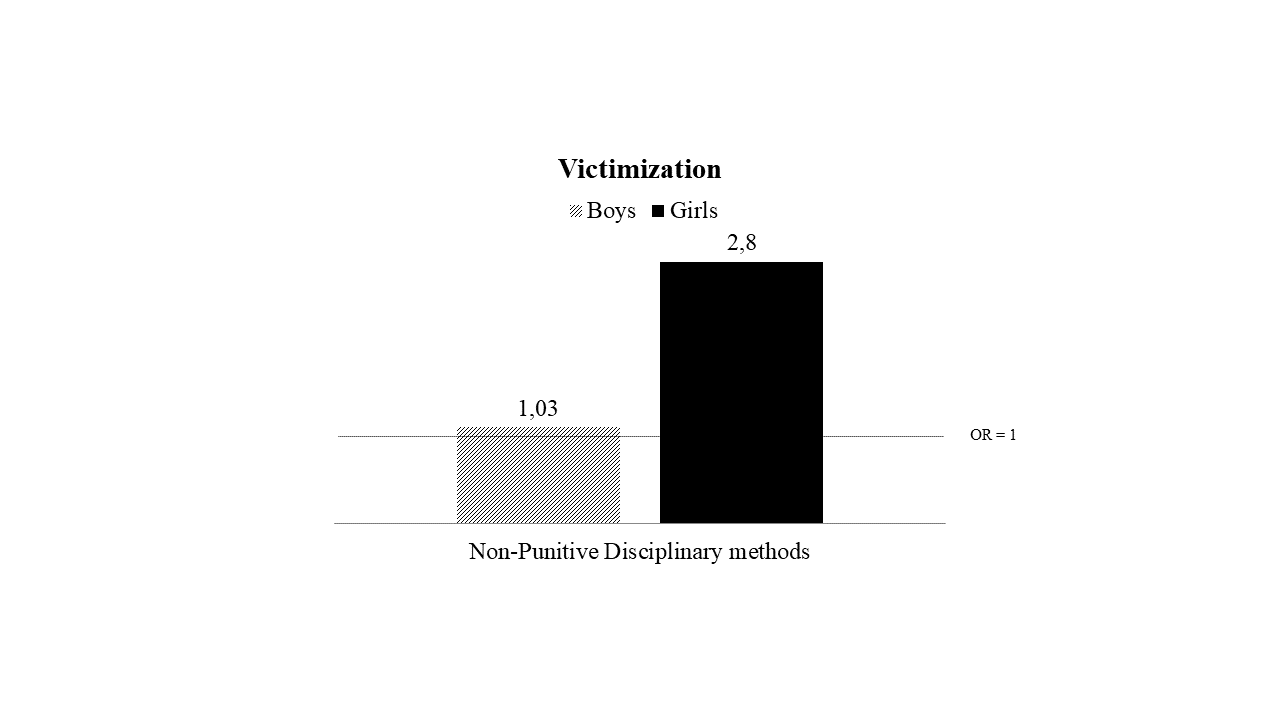


**Figure 2**

*Interaction Effects (Odds ratios) of Initial Perpetration Levels× School Assemblies and Playground Supervision on Post-Intervention Bullying Perpetration.*


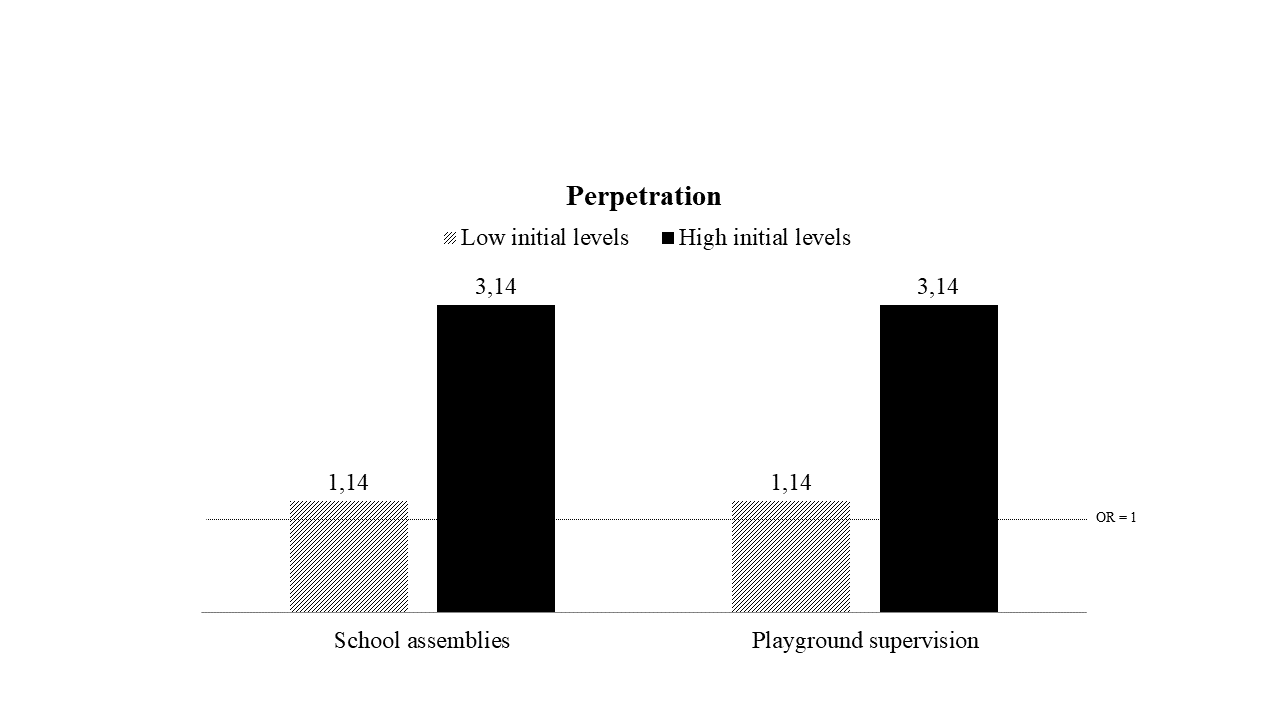


**S10.** Sensitivity Analysis

*Note.* Sensitivity analyses were performed for findings that were significant in the initial analyses. Analyses were performed by excluding trials from the original model one by one. In line with the data sharing agreement of this study, we do not indicate to which study the trial numbers specifically belonged.

**Table 1.** *Sensitivity Analyses For the Univariate Multilevel Regressions for the Entire Sample (i.e., Do Anti-Bullying Interventions Work?).*

|  |  | Coefficient | *SE* | *t* | Sig. | Exp (Coefficient) | 95% CI (coef.) |
| --- | --- | --- | --- | --- | --- | --- | --- |
|  |  |  |  |  |  |  | [LL, UL] |
| All trials | Victimization | -0.261 | 0.040 | -6.605 | <.001 | 0.770 | [0.71, 0.83] |
|  | Perpetration | -0.126 | 0.055 | -2.303 | .021 | 0.881 | [0.79, 0.98] |
| Without trial 1 | Victimization | -0.293 | .041 | -7.115 | .000 | 0.746 | [0.69, 0.81] |
|  | Perpetration | -0.210 | .059 | -3.541 | .000 | 0.811 | [0.72, 0.91] |
| Without trial 2 | Victimization | -0.264 | .040 | -6.671 | .000 | 0.768 | [0.71, 0.83] |
|  | Perpetration | -0.127 | .055 | -2.317 | .021 | 0.881 | [0.79, 0.98] |
| Without trial 3 | Victimization | -0.263 | .040 | -6.623 | .000 | 0.769 | [0.72, 0,83] |
|  | Perpetration | -0.124 | .055 | -2.256 | .024 | 0.883 | [0.79, 0.98] |
| Without trial 4 | Victimization | -0.268 | .040 | -6.695 | .000 | 0.765 | [0.71, 0.83] |
|  | Perpetration | -0.126 | .055 | -2.303 | .021 | 0.881 | [0.79, 0.98] |
| Without trial 5 | Victimization | -0.217 | .041 | -5.277 | .000 | 0.805 | [0.74, 0.87] |
|  | Perpetration | **-0.096** | **.057** | **-1.686** | **.092** | 0.909 | [0.81, 1,01] |
| Without trial 6 | Victimization | -0.235 | .045 | -5.252 | .000 | 0.790 | [0.72, 0.86] |
|  | Perpetration | -0.139 | .060 | -2.322 | .020 | 0.871 | [0.77, 0.98] |
| Without trial 7 | Victimization | -0.264 | .040 | -6.656 | .000 | 0.768 | [0.71, 0.83] |
|  | Perpetration | -0.114 | .055 | -2.053 | .040 | 0.893 | [0.80, 1.00] |
| Without trial 8 | Victimization | -0.261 | .040 | -6.605 | .000 | 0.770 | [0.71, 0.83] |
|  | Perpetration | -0.126 | .055 | -2.303 | .021 | 0.881 | [0.79, 0.98] |
| Without trial 9 | Victimization | -0.270 | .041 | -6.588 | .000 | 0.763 | [0.70, 0.83] |
|  | Perpetration | -0.139 | .059 | -2.490 | .013 | 0.870 | [0.78, 0.97] |
| Without trial 10 | Victimization | -0.283 | .048 | -5.901 | .000 | 0.754 | [0.69, 0.83] |
|  | Perpetration | **-.008** | **.063** | **-0.132** | **.985** | 0.992 | [0.88, 1,12] |
| Without trial 11 | Victimization | -0.255 | .044 | -5.744 | .000 | 0.775 | [0.71, 0.85] |
|  | Perpetration | -0.179 | .067 | -2.666 | .008 | 0.836 | [0.77, 0.95] |

*Note.* Main effects of interventions.

**Table 2.** *Sensitivity Analyses for Post-Intervention Victimization Model on Interaction Effects of Subgroup × Intervention Status.*

|  | **Victimization Model** | Coefficient | *SE* | *t* | Sig. | Exp (Coefficient) | 95% CI (coef.) |
| --- | --- | --- | --- | --- | --- | --- | --- |
|  |  |  |  |  |  |  | [LL, UL] |
| All trials | Initial victimization | 1.913 | 0.061 | 31.398 | <.001 | 6.775 | [6.01, 7.64] |
|  | Intervention | -0.202 | 0.049 | -4.145 | <.001 | 0.817 | [0.74, 0.90] |
|  | Initialvictimiz.* intervention | -0.168 | 0.081 | -2.065 | .039 | 0.845 | [0.72, 0.99] |
| Without trial 1 | Initial victimization | 1.972 | 0.065 | 30.401 | <.001 | 7.188 | [6.33, 8.16] |
|  | Intervention | -0.217 | 0.051 | -4.240 | <.001 | 0.805 | [0.73, 0.89] |
|  | Initialvictimiz.* intervention | -.216 | 0.085 | -2.528 | .011 | 0.806 | [0.68, 0.95] |
| Without trial 2 | Initial victimization | 1.912 | 0.061 | 31.339 | <.001 | 6.764 | [6.00, 7.65] |
|  | Intervention | -0.205 | 0.049 | -4.203 | <.001 | 0.814 | [0.74, 0.90] |
|  | Initialvictimiz.* intervention | -0.168 | 0.082 | -2.054 | .040 | 0.846 | [0.72, 0.99] |
| Without trial 3 | Initial victimization | 1.901 | 0.061 | 31.137 | <.001 | 6.694 | [5.94, 7.55] |
|  | Intervention | -0.208 | 0.0 | -4.258 | <.001 | 0.812 | [0.74, 0.89] |
|  | Initialvictimiz.* intervention | **-0.155** | **0.082** | **.901** | **.057** | 0.856 | [0.73, 1,00] |
| Without trial 4 | Initial victimization | 1.918 | 0.062 | 31.162 | <.001 | 6.807 | [6.03, 7.68] |
|  | Intervention | -0.206 | 0.049 | -4.162 | <.001 | 0.814 | [0.74, 0.90] |
|  | Initialvictimiz.* intervention | -0.178 | 0.082 | -2.161 | .031 | 0.837 | [0.71, 0.98] |
| Without trial 5 | Initial victimization | 1.883 | 0.064 | 29.499 | <.001 | 6.574 | [5.80, 7.45] |
|  | Intervention | -.162 | 0.051 | -3.203 | <.001 | 0.850 | [0.77, 0.94] |
|  | Initialvictimiz.* intervention | **-0.154** | **0.084** | **-1.827** | **.068** | 0.857 | [0.73, 1.01] |
| Without trial 6 | Initial victimization | 1.995 | 0.068 | 29.282 | <.001 | 7.353 | [6.34, 8.40] |
|  | Intervention | -.225 | 0.054 | -4.182 | <.001 | 0.798 | [0.72, 0.89] |
|  | Initialvictimiz.* intervention | **-0.032** | **0.096** | **-.331** | **.741** | 0.969 | [0.80, 1.17] |
| Without trial 7 | Initial victimization | 1.916 | 0.061 | 31.396 | <.001 | 6.792 | [6.03, 7.66] |
|  | Intervention | -0.204 | 0.049 | -4.169 | <.001 | 0.815 | [0.74, 0.90] |
|  | Initialvictimiz.* intervention | -0.170 | 0.082 | -2.085 | .037 | 0.843 | [0.72, 0.99] |
| Without trial 8 | Initial victimization | 1.913 | 0.061 | 31.398 | <.001 | 6.775 | [6.01, 7.64] |
|  | Intervention | -0.202 | 0.049 | -4.145 | <.001 | 0.817 | [0.74, 0.90] |
|  | Initialvictimiz.* intervention | -0.168 | 081 | -2.065 | .039 | 0.845 | [0.72, 0.99] |
| Without trial 9 | Initial victimization | 1.931 | 0.063 | 30.605 | <.001 | 6.897 | [6.10, 7.81] |
|  | Intervention | -0.213 | 0.051 | -4.179 | <.001 | 0.809 | [0.73, 0.89] |
|  | Initialvictimiz.* intervention | **-0.163** | **0.084** | **-1.934** | **.053** | 0.850 | [0.72, 1.00] |
| Without trial 10 | Initial victimization | 1.895 | 0.073 | 26.022 | <.001 | 6.653 | [5.77, 7.67] |
|  | Intervention | -0.201 | 0.060 | -3.358 | <.001 | 0.818 | [0.73, 0.92] |
|  | Initialvictimiz.* intervention | -0.219 | 0.097 | -2.261 | .024 | 0.804 | [0.67, 0.97] |
| Without trial 11 | Initial victimization | 1.795 | 0.068 | 26.376 | <.001 | 6.021 | [5.27, 6.88] |
|  | Intervention | -0.188 | 0.055 | -3.419 | <.001 | 0.828 | [0.74, 0.92] |
|  | Initialvictimiz.* intervention | -0.186 | 0.090 | -2.059 | .040 | 0.830 | [0.70, 0.99] |

**Table 3.** *Sensitivity Analyses for Post-Intervention Perpetration Model on Interaction Effects of Subgroup × Intervention Status.*

|  | **Perpetration Model** | Coefficient | *SE* | *t* | Sig. | Exp (Coefficient) | 95% CI (coef.) |
| --- | --- | --- | --- | --- | --- | --- | --- |
|  |  |  |  |  |  |  | [LL, UL] |
| All trials | Age | -0.022 | 0.115 | -0.191 | .848 | 0.978 | [0.78, 1.23] |
|  | Intervention | -0.342 | 0.091 | -3.756 | <.001 | 0.710 | [0.59, 0.85] |
|  | Age * intervention | 0.357 | 0.121 | 2.954 | .003 | 1.429 | [1.13, 1.81] |
| Without trial 1 | Age | -0.083 | 0.156 | -0.532 | .595 | 0.920 | [0.68, 1.25] |
|  | Intervention | -0.435 | 0.100 | -4.363 | <.001 | 0.647 | [-0.53, 0.78] |
|  | Age * intervention | 0.335 | 0.133 | 2.517 | .012 | 1.398 | [1.08, 1.82] |
| Without trial 2 | Age | -0.001 | 0.114 | -0.008 | .993 | 0.999 | [0.80, 1.25] |
|  | Intervention | -0.343 | 0.091 | -3.771 | <.001 | 0.709 | [0.59, 0.85] |
|  | Age * intervention | 0.358 | 0.121 | 2.962 | .003 | 1.430 | [1.13, 1.81] |
| Without trial 3 | Age | -0.023 | 0.115 | -0.196 | .844 | 0.978 | [0.78, 1.22] |
|  | Intervention | -0.336 | 0.091 | -3.678 | <.001 | 0.715 | [0.69, 0.86] |
|  | Age * intervention | 0.350 | 0.121 | 2.897 | .004 | 1.420 | [1.12, 1.80] |
| Without trial 4 | Age | -0.022 | 0.115 | -0.191 | .848 | 0.978 | [0.78, 1.23] |
|  | Intervention | -0.342 | 0.091 | -3.756 | <.001 | 0.710 | [0.59, 0.85] |
|  | Age * intervention | 0.357 | 0.121 | 2.954 | .003 | 1.429 | [1.13, 1.81] |
| Without trial 5 | Age | -0.000 | 0.116 | -0.000 | 1.000 | 1.000 | [0.80, 1.26] |
|  | Intervention | -0.287 | 0.101 | -2.855 | .004 | 0.750 | [0.62, 0.91] |
|  | Age * intervention | 0.299 | 0.128 | 2.338 | .019 | 1.349 | [1.05, 1.73] |
| Without trial 6 | Age | -0.022 | 0.115 | -0.191 | .848 | 0.978 | [0.78, 1.23] |
|  | Intervention | -0.342 | 0.091 | -3.756 | <.001 | 0.710 | [0.59, 0.85] |
|  | Age * intervention | 0.357 | 0.121 | 2.954 | .003 | 1.429 | [1.13, 1.81] |
| Without trial 7 | Age | -0.042 | 0.115 | -0.360 | .719 | 0.959 | [0.77, 1.20] |
|  | Intervention | -0.342 | 0.091 | -3.752 | <.001 | 0.710 | [0.59, 0.85] |
|  | Age * intervention | 0.390 | 0.122 | 3.206 | 0.001 | 1.478 | [1.16, 1.88] |
| Without trial 8 | Age | -0.022 | 0.115 | -0.191 | .848 | 0.978 | [0.78, 1.23] |
|  | Intervention | -0.342 | 0.091 | -3.756 | <.001 | 0.710 | [0.59, 0.85] |
|  | Age * intervention | 0.357 | 0.121 | 2.954 | .003 | 1.429 | [1.13, 1.81] |
| Without trial 9 | Age | -0.049 | 0.115 | -0.427 | .670 | 0.952 | [0.76, 1.19] |
|  | Intervention | -0.405 | 0.096 | -4.203 | <.001 | 0.667 | [0.55, 0.81] |
|  | Age * intervention | 0.419 | 0.125 | 3.360 | .001 | 1.521 | [1.19, 1.94] |
| Without trial 10 | Age | 0.126 | 0.156 | 0.807 | .420 | 1.134 | [0.83, 1.54] |
|  | Intervention | -0.161 | 0.138 | -1.170 | .242 | 0.851 | [0.65, 1.12] |
|  | **Age * intervention** | **0.225** | **0.161** | **1.404** | **.160** | 1.254 | [0.91, 1.72] |
| Without trial 11 | Age | -0.048 | 0.128 | -0.376 | .707 | 0.953 | [0.74, 1.23] |
|  | Intervention | -0.339 | 0.091 | -3.741 | <.001 | 0.713 | [0.59, 0.85] |
|  | Age * intervention | 0.430 | 0.171 | 2.514 | .012 | 1.537 | [1.10, 2.15] |

**Table 4.** *Sensitivity Analyses for Main Effects of Intervention Components on Post-Intervention Bullying Victimization*

|  | **Victimization Model** | Coefficient | *SE* | *t* | Sig. | Exp (Coefficient) | 95% CI (coef.) |
| --- | --- | --- | --- | --- | --- | --- | --- |
|  |  |  |  |  |  |  | [LL, UL] |
| All trials | Disciplinary methods |  |  |  |  |  |  |
|  | *Non-punitive [=2]* | 0.879 | 0.402 | 2.187 | .029 | 2.408 | [1.10, 5.29] |
|  | *Non-punitive and punitive [=3]* | 0.466 | 0.381 | 1.223 | .221 | 1.594 | [0.76, 3.37] |
| Without trial 1 | Disciplinary methods |  |  |  |  |  |  |
|  | *Non-punitive* | **0.649** | **0.410** | **1.584** | **0.11** | 1.913 | [0.86, 4.27] |
|  | *Non-punitive and punitive* | 0.469 | 0.359 | 1.308 | 0.19 | 1.599 | [0.79, 3.23] |
| Without trial 2 | Disciplinary methods |  |  |  |  |  |  |
|  | *Non-punitive* | **0.703** | **0.452** | **1.555** | **0.12** | 2.021 | [0.83, 4.90] |
|  | *Non-punitive and punitive* | 0.291 | 0.433 | 0.671 | 0.50 | 1.338 | [0.57, 3.13] |
| Without trial 3 | Disciplinary methods |  |  |  |  |  |  |
|  | *Non-punitive* | 1.125 | 0.447 | 2.518 | 0.01 | 3.080 | [1.28, 7.39] |
|  | *Non-punitive and punitive* | 0.712 | 0.429 | 1.661 | 0.097 | 2.039 | [0.88, 4.72] |
| Without trial 4 | Disciplinary methods |  |  |  |  |  |  |
|  | *Non-punitive* | 1.077 | 0.420 | 2.567 | 0.010 | 2.937 | [1.29, 6.69] |
|  | *Non-punitive and punitive* | 0.468 | 0.370 | 1.266 | 0.206 | 1.597 | [0.77, 3.30] |
| Without trial 5 | Disciplinary methods |  |  |  |  |  |  |
|  | *Non-punitive* | 0.877 | 0.424 | 2.069 | 0.039 | 2.405 | [1.05, 5.52] |
|  | *Non-punitive and punitive* | 0.521 | 0.421 | 1.239 | 0.216 | 1.684 | [0.74, 3.85] |
| Without trial 6 | Disciplinary methods |  |  |  |  |  |  |
|  | *Non-punitive* | **0.880** | **0.476** | **1.846** | **0.065** | 2.310 | [0.95, 6.13] |
|  | *Non-punitive and punitive* | 0.446 | 0.411 | 1.084 | 0.278 | 1.562 | [0.70, 3.50] |
| Without trial 7 | Disciplinary methods |  |  |  |  |  |  |
|  | *Non-punitive* | **0.784** | **0.508** | **1.543** | **0.123** | 2.190 | [0.81, 5.98] |
|  | *Non-punitive and punitive* | 0.372 | 0.490 | 0.759 | 0.448 | 1.451 | [0.55, 3.79] |
| Without trial 8 | Disciplinary methods |  |  |  |  |  |  |
|  | *Non-punitive* | 0.879 | 0.402 | 2.187 | 0.029 | 2.404 | [1.10, 5.29] |
|  | *Non-punitive and punitive* | 0.466 | 0.381 | 1.223 | 0.221 | 1.594 | [0.75, 3.37] |
| Without trial 9 | Disciplinary methods |  |  |  |  |  |  |
|  | *Non-punitive* | 0.870 | 0.429 | 2.027 | 0.043 | 2.386 | [1.03, 5.53] |
|  | *Non-punitive and punitive* | 0.423 | 0.426 | 0.991 | 0.322 | 1.526 | [0.66, 3.53] |
| Without trial 10 | Disciplinary methods |  |  |  |  |  |  |
|  | *Non-punitive* | 0.902 | 0.360 | 2.506 | 0.012 | 2.464 | [1.22, 4.99] |
|  | *Non-punitive and punitive* | 0.301 | 0.358 | 0.840 | 0.401 | 1.351 | [0.67, 2.73] |
| Without trial 11 | Disciplinary methods |  |  |  |  |  |  |
|  | *Non-punitive* | 0.911 | 0.374 | 2.434 | 0.015 | 2.487 | [1.19, 5.18] |
|  | *Non-punitive and punitive* | 0.639 | 0.372 | 1.714 | 0.087 | 1.894 | [0.91, 3.93] |

**Table 5.** *Sensitivity Analyses for Main Effects of Intervention Components on Post-Intervention Bullying Perpetration*

|  | **Perpetration Model** | Coefficient | *SE* | *t* | Sig. | Exp (Coefficient) | 95% CI (coef.) | |
| --- | --- | --- | --- | --- | --- | --- | --- | --- |
|  |  |  |  |  |  |  | [LL, UL] | |
| All trials | Disciplinary methods |  |  |  |  |  |  |  |
|  | *Non-punitive* | 1.782 | 0.776 | 2.297 | .022 | 5.940 | [1.30, 27.16] | |
|  | *Non-punitive and punitive* | 0.894 | 0.690 | 1.296 | .195 | 2.444 | [0.63, 9.44] | |
| Without trial 1 | Disciplinary methods |  |  |  |  |  |  |  |
|  | *Non-punitive* | **0.491** | **0.345** | **1.424** | **0.154** | 1.635 | [0.38, 3.22] | |
|  | *Non-punitive and punitive* | 0.407 | 0.309 | 1.317 | 0.188 | 1.503 | [0.82, 2.76] | |
| Without trial 2 | Disciplinary methods |  |  |  |  |  |  | |
|  | *Non-punitive* | **1.203** | **0.695** | **1.731** | **0.083** | 3.311 | [0.85, 13.01] | |
|  | *Non-punitive and punitive* | 0.320 | 0.632 | 0.506 | 0.613 | 1.377 | [0.40, 4.75] | |
| Without trial 3 | Disciplinary methods |  |  |  |  |  |  | |
|  | *Non-punitive* | **1.493** | **0.765** | **1.952** | **0.051** | 4.449 | [0.99, 19.92] | |
|  | *Non-punitive and punitive* | 0.606 | 0.689 | 0.880 | 0.379 | 1.833 | [0.48, 7.07] | |
| Without trial 4 | Disciplinary methods |  |  |  |  |  |  | |
|  | *Non-punitive* | - | - |  | - |  |  | |
|  | *Non-punitive and punitive* | - | - |  | - |  |  | |
| Without trial 5 | Disciplinary methods |  |  |  |  |  |  | |
|  | *Non-punitive* | 1.918 | 0.919 | 2.086 | 0.037 | 6.804 | [1.12, 41.25] | |
|  | *Non-punitive and punitive* | 1.037 | 0.850 | 1.220 | 0.222 | 2.821 | [0.54, 14.92] | |
| Without trial 6 | Disciplinary methods |  |  |  |  |  |  | |
|  | *Non-punitive* | 1.484 | 0.242 | 6.140 | 0.000 | 4.410 | [2.85, 7.08] | |
|  | *Non-punitive and punitive* | 0.201 | 0.218 | 0.923 | 0.356 | 1.223 | [0.80, 1.88] | |
| Without trial 7 | Disciplinary methods |  |  |  |  |  |  | |
|  | *Non-punitive* | **15.173** | **479.924** | **0.032** | **0.976** | 1612743.028 | 0 | |
|  | *Non-punitive and punitive* | 14.293 | 479.924 |  | 0.975 | 3887090.922 | 0 | |
| Without trial 8 | Disciplinary methods |  |  |  |  |  |  | |
|  | *Non-punitive* | - | - |  | - |  |  | |
|  | *Non-punitive and punitive* | - | - | - | - |  |  | |
| Without trial 9 | Disciplinary methods |  |  |  |  |  |  | |
|  | *Non-punitive* | 1.897 | 0.897 | 2.115 | 0.034 | 6.664 | [1.15, 38.65] | |
|  | *Non-punitive and punitive* | 1.091 | 0.829 | 1.316 | 0.188 | 2.977 | [0.59, 15.11] | |
| Without trial 10 | Disciplinary methods |  |  |  |  |  |  | |
|  | *Non-punitive* | 1.941 | 0.924 | 2.101 | 0.036 | 6.964 | [1.14, 42.56] | |
|  | *Non-punitive and punitive* | 1.007 | 0.855 | 1.178 | 0.239 | 2.727 | [0.51, 14.62] | |
| Without trial 11 | Disciplinary methods |  |  |  |  |  |  | |
|  | *Non-punitive* | 1.964 | 0.917 | 2.142 | 0.032 | 7.125 | [1.18, 42.97] | |
|  | *Non-punitive and punitive* | 0.964 | 0.849 | 1.136 | 0.256 | 2.623 | [0.50, 13.84] | |

**Table 6***. Sensitivity Analyses for the Victimization Model on Interaction Effects of Sex x Intervention Components for Post-Intervention Bullying Victimization*

|  | **Victimization Model** | Coefficient | *SE* | *t* | Sig. | Exp (Coefficient) | 95% CI (coef.) |
| --- | --- | --- | --- | --- | --- | --- | --- |
|  |  |  |  |  |  |  | [LL, UL] |
| All trials | Sex | 1.008 | 0.436 | -2.315 | .021 | 0.365 | [0.16, 0.86] |
|  | Non punitive | 0.456 | 0.426 | 1.068 | .285 | 1.577 | [0.68, 3.64] |
|  | Non punitive and punitive | 0.179 | 0.406 | 0.440 | .660 | 1.196 | [0.54, 2.65] |
|  | Non-punitive * sex | 1.029 | 0.444 | 2.316 | .021 | 2.799 | [1.17, 6.69] |
|  | Non-punitive and punitive * sex | 0.740 | 0.441 | 1.679 | .093 | 2.097 | [0.88, 4.98] |
| Without trial 1 | Sex | -1.010 | .436 | -2.318 | .020 | 0.364 | [0.15, 0.86] |
|  | Non-punitive | 0.199 | .434 | .457 | .648 | 1.220 | [0.52, 2.86] |
|  | Non-punitive and punitive | 0.182 | .385 | 0.471 | .638 | 1.199 | [0.56, 2.55] |
|  | Non-punitive * sex | 1.085 | .446 | 2.433 | .015 | 2.959 | [1.24, 7.09] |
|  | Non-punitive and punitive * sex | 0.742 | .441 | 1.683 | .092 | 2.100 | [0.86, 4.98] |
| Without trial 2 | Sex | -1.144 | .492 | -2.326 | .020 | 0.319 | [0.12, 0.84] |
|  | Non-punitive | 0.267 | .477 | 0.559 | .576 | 1.305 | [0.51, 3.32] |
|  | Non-punitive and punitive | -0.010 | .457 | -0.022 | .982 | 0.990 | [0.40, 2.43] |
|  | Non-punitive * sex | 1.165 | .499 | 2.332 | .020 | 3.205 | [1.20, 8.53] |
|  | Non-punitive and punitive * sex | 0.876 | .496 | 1.764 | .078 | 2.401 | [0.91, 6.35] |
| Without trial 3 | Sex | -1.297 | .528 | -2.455 | .014 | 0.273 | [0.10, 0.77] |
|  | Non-punitive | 0.610 | .471 | 1.295 | .195 | 1.841 | [0.73, 4.64] |
|  | Non-punitive and punitive | 0.333 | .453 | .736 | .462 | 1.396 | [0.57, 3.39] |
|  | Non-punitive * sex | 1.317 | .535 | 2.461 | .014 | 3.734 | [1.31, 10.66] |
|  | Non-punitive and punitive * sex | 1.029 | .532 | 1.932 | .052 | 2.797 | [0.99, 7.94] |
| Without trial 4 | Sex | -1.009 | .436 | -2.316 | .021 | 0.365 | [0.15, 0.86] |
|  | Non-punitive | .646 | .443 | 1.458 | .145 | 1.908 | [0.80, 4.55] |
|  | Non-punitive and punitive | .181 | .392 | .458 | .647 | 1.198 | [0.55, 2.60] |
|  | Non-punitive * sex | 1.046 | .445 | .351 | .019 | 2.947 | [1.19, 6.81] |
|  | Non-punitive and punitive * sex | 0.741 | .441 | 1.680 | .093 | 2.097 | [0.88, 4.98] |
| Without trial 5 | Sex | -1.007 | .436 | -2.312 | .021 | 0.365 | [0.15, 0.86] |
|  | Non-punitive | .454 | .447 | 1.017 | .309 | 1.575 | [0.66, 3.78] |
|  | Non-punitive and punitive | .239 | .443 | .540 | .589 | 1.270 | [0.54, 3.03] |
|  | Non-punitive * sex | 1.028 | .444 | 2.313 | .021 | 2.795 | [1.17, 6.68] |
|  | Non-punitive and punitive * sex | .0731 | .441 | 1.657 | .098 | 2.077 | [0.88, 4.93] |
| Without trial 6 | Sex | -1.020 | .438 | -2.326 | .020 | 0.361 | [0.15, 0.85] |
|  | Non-punitive | .591 | .503 | 1.174 | .240 | 1.805 | [0.67, 4.84] |
|  | Non-punitive and punitive | .151 | .434 | .347 | .729 | 1.163 | [0.50, 2.72] |
|  | Non-punitive * sex | **.757** | **.476** | **1.589** | **.112** | 2.131 | [0.84, 5.42] |
|  | Non-punitive and punitive * sex | .765 | .444 | 1.705 | .088 | 2.131 | [0.89, 5.08] |
| Without trial 7 | Sex | -.273 | .641 | -.425 | .671 | 0.761 | [0.22, 2.67] |
|  | Non-punitive | .632 | .592 | 1.066 | .286 | 1.881 | [0.59, 6.01] |
|  | Non-punitive and punitive | .355 | .576 | .616 | .538 | 1.426 | [0.46, 4.42] |
|  | Non-punitive * sex | **.293** | **.647** | **.454** | **.650** | 1.341 | [0.38, 4.76] |
|  | Non-punitive and punitive * sex | .004 | .644 | .007 | .995 | 1.004 | [0.28, 3.55] |
| Without trial 9 | Sex | -1.009 | .436 | -2.313 | .021 | 0.365 | [0.15, 0.86] |
|  | Non-punitive | .446 | .452 | .986 | .324 | 1.561 | [0.64, 3.79] |
|  | Non-punitive and punitive | .147 | .448 | .328 | .743 | 1.158 | [0.48, 2.79] |
|  | Non-punitive * sex | 1.030 | .445 | 2.317 | .021 | 2.802 | [1.17, 6.70] |
|  | Non-punitive and punitive * sex | .711 | .442 | 1.610 | .107 | 2.037 | [0.86, 4.84] |
| Without trial 10 | Sex | -1.006 | .435 | -2.315 | .021 | 0.366 | [0.16, 0.86] |
|  | Non-punitive | .483 | .386 | 1.251 | .211 | 1.621 | [0.76, 3.45] |
|  | Non-punitive and punitive | .062 | .384 | .161 | .872 | 1.064 | [0.50, 2.26] |
|  | Non-punitive * sex | 1.025 | .443 | 2.312 | .021 | 2.787 | [1.17, 6.65] |
|  | Non-punitive and punitive * sex | .627 | .445 | 1.410 | .159 | 1.873 | [0.78, 4.48] |
| Without trial 11 | Sex | -1.003 | .434 | -2.311 | .021 | 0.367 | [0.16, 0.86] |
|  | Non-punitive | .492 | .399 | 1.233 | .218 | 1.636 | [0.75, 3.58] |
|  | Non-punitive and punitive | .295 | .397 | .744 | .457 | 1.344 | [0.62, 2.93] |
|  | Non-punitive * sex | 1.020 | .443 | 2.303 | .021 | 2.773 | [1.16, 6.61] |
|  | Non-punitive and punitive * sex | .864 | .442 | 1.956 | .050 | 2.373 | [1.00, 5.64] |

**Table 7.** *Sensitivity Analyses in Perpetration Model for Interaction Effects of Initial Level of Perpetration (ISP) x Intervention Components for Post-Intervention Perpetration.*

|  | **Perpetration Model** | Coefficient | *SE* | *t* | Sig. | Exp (Coefficient) | 95% CI (coef.) |
| --- | --- | --- | --- | --- | --- | --- | --- |
|  |  |  |  |  |  |  | [LL, UL] |
| All trials | ISP | 1.105 | 0.230 | 4.800 | <.001 | 3.019 | [1.92, 4.74] |
|  | School assemblies | -0.014 | 0.741 | -0.019 | .985 | 0.986 | [0.23, 4.21] |
|  | School assemblies * ISP | 1.144 | 0.247 | 4.632 | <.001 | 3.139 | [1.93, 5.09] |
|  | ISP | 1.105 | 0.230 | 4.800 | <.001 | 3.019 | [1.92, 4.74] |
|  | Playground supervision | -0.014 | 0.741 | -0.019 | .985 | 0.986 | [0.23, 4.21] |
|  | Playground supervision * ISP | 1.144 | 0.247 | 4.632 | <.001 | 3.139 | [1.93, 5.09] |
| Without trial 1 | ISP | 0.352 | 0.986 | 0.357 | 0.721 | 1.422 | [0.21, 9.82] |
|  | School assemblies | 0.099 | 0.225 | 0.443 | 0.658 | 1.105 | [0.71, 1.72] |
|  | School assemblies * ISP | **1.588** | **0.989** | **1.606** | **0.108** | 4.896 | [0.70, 34.03] |
|  | ISP | 0.352 | 0.986 | 0.357 | 0.721 | 1.422 | [0.21, 9.82] |
|  | Playground supervision | 0.099 | 0.225 | 0.443 | 0.658 | 1.105 | [0.71, 1.72] |
|  | Playground supervision * ISP | **1.588** | **0.989** | **1.606** | **0.108** | 4.896 | [0.70, 34.03] |
| Without trial 2 | ISP | 1.113 | 0.230 | 4.838 | 0.000 | 3.044 | [1.94, 4.78] |
|  | School assemblies | -0.720 | 0.524 | -1.372 | 0.170 | 0.487 | [0.17, 1.36] |
|  | School assemblies * ISP | 1.136 | 0.247 | 4.606 | 0.000 | 3.116 | [1.92, 5.05] |
|  | ISP | 1.113 | 0.230 | 4.838 | 0.000 | 3.044 | [1.94, 4.78] |
|  | Playground supervision | -0.720 | 0.524 | -1.372 | 0.170 | 0.487 | [0.17, 1.36] |
|  | Playground supervision * ISP | 1.136 | 0.247 | 4.606 | 0.000 | 3.116 | [1.92, 5.05] |
| Without trial 3 | ISP | 1.102 | 0.230 | 4.795 | 0.000 | 3.012 | [1.92, 4.73] |
|  | School assemblies | -0.445 | 0.665 | -0.668 | 0.504 | 0.641 | [0.17, 2.36] |
|  | School assemblies * ISP | 1.146 | 0.247 | 4.648 | 0.000 | 3.147 | [1.94, 5.10] |
|  | ISP | 1.102 | 0.230 | 4.795 | 0.000 | 3.012 | [1.92, 4.73] |
|  | Playground supervision | -0.445 | 0.665 | -0.668 | 0.504 | 0.641 | [0.17, 2.36] |
|  | Playground supervision * ISP | 1.146 | 0.247 | 4.648 | 0.000 | 3.147 | [1.94, 5.10] |
| Without trial 4 | ISP | - | - |  | - |  |  |
|  | School assemblies | - | - |  | - |  |  |
|  | School assemblies * ISP | - | - |  | - |  |  |
|  | ISP | - | - |  | - |  |  |
|  | Playground supervision | - | - |  | - |  |  |
|  | Playground supervision * ISP | - | - |  | - |  |  |
| Without trial 5 | ISP | 1.100 | 0.230 | 4.777 | 0.000 | 3.004 | [1.91, 4.72] |
|  | School assemblies | 0.106 | 0.899 | 0.117 | 0.906 | 1.111 | [0.19, 6.47] |
|  | School assemblies * ISP | 1.159 | 0.248 | 4.673 | 0.000 | 3.185 | [1.96, 5.18] |
|  | ISP | 1.100 | 0.230 | 4.777 | 0.000 | 3.004 | [1.91, 4.72] |
|  | Playground supervision | 0.106 | 0.899 | 0.117 | 0.906 | 1.111 | [0.19, 6.47] |
|  | Playground supervision * ISP | 1.159 | 0.248 | 4.673 | 0.000 | 3.185 | [1.96, 5.18] |
| Without trial 6 | ISP | 1.100 | 0.230 | 4.777 | 0.000 | 3.005 | [1.91, 4.72] |
|  | School assemblies | 0.012 | 0.899 | 0.014 | 0.989 | 1.012 | [0.17, 5.89] |
|  | School assemblies * ISP | 1.388 | 0.253 | 5.492 | 0.000 | 4.008 | [2.44, 6.58] |
|  | ISP | 1.100 | 0.230 | 4.777 | 0.000 | 3.005 | [1.91, 4.72] |
|  | Playground supervision | 0.012 | 0.899 | 0.014 | 0.989 | 1.012 | [0.17, 5.89] |
|  | Playground supervision * ISP | 1.388 | 0.253 | 5.492 | 0.000 | 4.008 | [2.44, 6.58] |
| Without trial 7 | ISP | 1.158 | 0.240 | 4.826 | 0.000 | 3.182 | [1.99, 5.09] |
|  | School assemblies | 0.290 | 0.989 | 0.293 | 0.769 | 1.336 | [0.19, 9.29] |
|  | School assemblies * ISP | 1.091 | 0.256 | 4.264 | 0.000 | 2.978 | [1.80, 4.92] |
|  | ISP | 1.158 | 0.240 | 4.826 | 0.000 | 3.182 | [1.99, 5.09] |
|  | Playground supervision | 0.290 | 0.989 | 0.293 | 0.769 | 1.336 | [0.19, 9.29] |
|  | Playground supervision * ISP | 1.091 | 0.256 | 4.264 | 0.000 | 2.978 | [1.80, 4.92] |
| Without trial 8 | ISP | - | - |  | - |  |  |
|  | School assemblies | - | - |  | - |  |  |
|  | School assemblies * ISP | - | - |  | - |  |  |
|  | ISP | - | - |  | - |  |  |
|  | Playground supervision | - | - |  | - |  |  |
|  | Playground supervision * ISP | - | - |  | - |  |  |
| Without trial 9 | ISP | 1.100 | 0.230 | 4.778 | 0.000 | 3.005 | [1.91, 4.72] |
|  | School assemblies | 0.148 | 0.889 | 0.167 | 0.868 | 1.160 | [0.20, 6.63] |
|  | School assemblies * ISP | 1.154 | 0.247 | 4.667 | 0.000 | 3.169 | [1.95, 5.15] |
|  | ISP | 1.100 | 0.230 | 4.778 | 0.000 | 3.005 | [1.91, 4.72] |
|  | Playground supervision | 0.148 | 0.889 | 0.167 | 0.868 | 1.160 | [0.20, 6.63] |
|  | Playground supervision * ISP | 1.154 | 0.247 | 4.667 | 0.000 | 3.169 | [1.95, 5.15] |
| Without trial 10 | ISP | 1.100 | 0.230 | 4.776 | 0.000 | 3.003 | [1.91, 4.72] |
|  | School assemblies | 0.101 | 0.902 | 0.112 | 0.911 | 1.106 | [0.19, 6.49] |
|  | School assemblies * ISP | 1.068 | 0.252 | 4.247 | 0.000 | 2.910 | [1.79, 4.77] |
|  | ISP | 1.100 | 0.230 | 4.776 | 0.000 | 3.003 | [1.91, 4.72] |
|  | Playground supervision | 0.101 | 0.902 | 0.112 | 0.911 | 1.106 | [0.19, 6.49] |
|  | Playground supervision * ISP | 1.068 | 0.252 | 4.247 | 0.000 | 2.910 | [1.79, 4.77] |
| Without trial 11 | ISP | 1.100 | 0.230 | 4.776 | 0.000 | 3.003 | [1.91, 4.72] |
|  | School assemblies | 0.103 | 0.901 | 0.114 | 0.909 | 1.108 | [0.19, 6.48] |
|  | School assemblies * ISP | 0.934 | 0.259 | 3.606 | 0.000 | 2.545 | [1.53, 4.23] |
|  | ISP | 1.100 | 0.230 | 4.776 | 0.000 | 3.003 | [1.91, 4.72] |
|  | Playground supervision | 0.103 | 0.901 | 0.114 | 0.909 | 1.108 | [0.19, 6.48] |
|  | Playground supervision * ISP | 0.934 | 0.259 | 3.606 | 0.000 | 2.545 | [1.53, 4.23] |

1. Juvonen et al., (2016), Kärnä et al., (2011), Salmivalli et al., (2005) share the same dataset. They were all willing to contribute to our IPD and thus are referred to individually but their data were only included once. [↑](#footnote-ref-2)
